# Antibody responses to a suite of novel serological markers for malaria surveillance demonstrate strong correlation with clinical and parasitological infection across seasons and transmission settings in The Gambia

**DOI:** 10.1186/s12916-020-01724-5

**Published:** 2020-09-25

**Authors:** Lindsey Wu, Julia Mwesigwa, Muna Affara, Mamadou Bah, Simon Correa, Tom Hall, Susheel K. Singh, James G. Beeson, Kevin K. A. Tetteh, Immo Kleinschmidt, Umberto D’Alessandro, Chris Drakeley

**Affiliations:** 1 Faculty of Infectious Tropical Diseases, Department of Infection Biology, London School of Hygiene and Tropical Medicine (LSHTM), London, WC1E 7HT UK; 2 Medical Research Council Unit The Gambia at London School of Hygiene and Tropical Medicine, Fajara, The Gambia; 3 Bernhard Nocht Institute for Tropical Medicine (BNITM), Arusha, Tanzania; 4 St. George’s University of London (SGUL), London, UK; 5 Department for Congenital Disorders, Statens Serum Institut, Copenhagen, Denmark; 6 Centre for Medical Parasitology at Department of Immunology and Microbiology, University of Copenhagen, Copenhagen, Denmark; 7 Burnet Institute, Melbourne, Australia; 8 Central Clinical School, Monash University, Melbourne, Victoria Australia; 9 Department of Medicine, University of Melbourne, Melbourne, Victoria Australia; 10 Faculty of Epidemiology and Population Health, Department of Infectious Disease Epidemiology, London School of Hygiene and Tropical Medicine (LSHTM), London, WC1E 7HT UK; 11 School of Pathology, Wits Institute for Malaria Research, Faculty of Health Science, University of Witwatersrand, Johannesburg, South Africa

**Keywords:** Malaria, Serology, Surveillance

## Abstract

**Background:**

As malaria transmission declines, sensitive diagnostics are needed to evaluate interventions and monitor transmission. Serological assays measuring malaria antibody responses offer a cost-effective detection method to supplement existing surveillance tools.

**Methods:**

A prospective cohort study was conducted from 2013 to 2015 in 12 villages across five administrative regions in The Gambia. Serological analysis included samples from the West Coast Region at the start and end of the season (July and December 2013) and from the Upper River Region in July and December 2013 and April and December 2014. Antigen-specific antibody responses to eight *Plasmodium falciparum* (*P. falciparum*) antigens—Etramp5.Ag1, GEXP18, HSP40.Ag1, Rh2.2030, EBA175 RIII-V, *Pf*MSP1_19_, *Pf*AMA1, and *Pf*GLURP.R2—were quantified using a multiplexed bead-based assay. The association between antibody responses and clinical and parasitological endpoints was estimated at the individual, household, and population level.

**Results:**

Strong associations were observed between clinical malaria and concurrent sero-positivity to Etramp5.Ag1 (aOR 4.60 95% CI 2.98–7.12), *Pf*MSP1_19_ (aOR 4.09 95% CI 2.60–6.44), *Pf*AMA1 (aOR 2.32 95% CI 1.40–3.85), and *Pf*GLURP.R2 (aOR 3.12, 95% CI 2.92–4.95), while asymptomatic infection was associated with sero-positivity to all antigens. Village-level sero-prevalence amongst children 2–10 years against Etramp5.Ag1, HSP40.Ag1, and *Pf*MSP1_19_ showed the highest correlations with clinical and *P. falciparum* infection incidence rates. For all antigens, there were increased odds of asymptomatic *P. falciparum* infection in subjects residing in a compound with greater than 50% sero-prevalence, with a 2- to 3-fold increase in odds of infection associated with Etramp5.Ag1, GEXP18, Rh2.2030, *Pf*MSP1_19_, and *Pf*AMA1. For individuals residing in sero-positive compounds, the odds of clinical malaria were reduced, suggesting a protective effect.

**Conclusions:**

At low transmission, long-lived antibody responses could indicate foci of malaria transmission that have been ongoing for several seasons or years. In settings where sub-patent infections are prevalent and fluctuate below the detection limit of polymerase chain reaction (PCR), the presence of short-lived antibodies may indicate recent infectivity, particularly in the dry season when clinical cases are rare. Serological responses may reflect a persistent reservoir of infection, warranting community-targeted interventions if individuals are not clinically apparent but have the potential to transmit. Therefore, serological surveillance at the individual and household level may be used to target interventions where there are foci of asymptomatically infected individuals, such as by measuring the magnitude of age-stratified antibody levels or identifying areas with clustering of above-average antibody responses across a diverse range of serological markers.

## Background

As malaria transmission declines, the prevalence of infection becomes increasingly heterogeneous and focal. In low transmission settings, highly sensitive diagnostics are needed to measure subtle changes in malaria incidence, evaluate the effectiveness of community-based interventions, and monitor potential re-introduction after elimination. Evidence suggests that infections below the detection limit of microscopy and rapid diagnostic tests (RDTs) contribute to on-going transmission, but the magnitude of their public health importance is yet to be determined [1–3]. Nucleic acid amplification tests (NAATs), such as ultra-sensitive quantitative polymerase chain reaction (qPCR), can detect less than 1 parasite per microlitre [4, 5], but are still sensitive to fluctuations in parasite density during the course of an infection.

Serological assays measuring antibody responses may be a more stable diagnostic alternative, offering a cost-effective detection method to supplement existing surveillance tools. A number of studies in The Gambia have observed spatio-temporal variations in antibody responses to malaria over time [6, 7], with similar observations in Tanzania [8], Equatorial Guinea [9], and South Africa [10]. The majority of these studies measure long-lived antibody responses, which can persist for years in the absence of re-infection. By contrast, new serological markers of recent malaria exposure have been developed based on antigens that elicit shorter-lived antibody responses and may be capable of measuring changes in malaria transmission dynamics over periods as short as 1–2 years [11, 12]. These markers require validation with population-representative data from a range of endemic settings, and longitudinal cohort studies provide a unique opportunity for a multi-metric assessment of transmission dynamics using clinical, parasitological, and serological endpoints.

Using a suite of novel serological markers of malaria exposure, this study aimed to determine the correlation of antibody responses with clinical and parasitological endpoints at the individual, household, and population level in rural Gambia over 2 years and estimate the strength of association between serological markers and gold standard metrics of active malaria infection. These findings can support the application of serology in measuring recent malaria transmission and allow selection of markers most suitable for use in future surveillance, particularly for reactive detection strategies in pre- and post-elimination settings.

## Methods

### Data and sampling

**Fig. 1:**
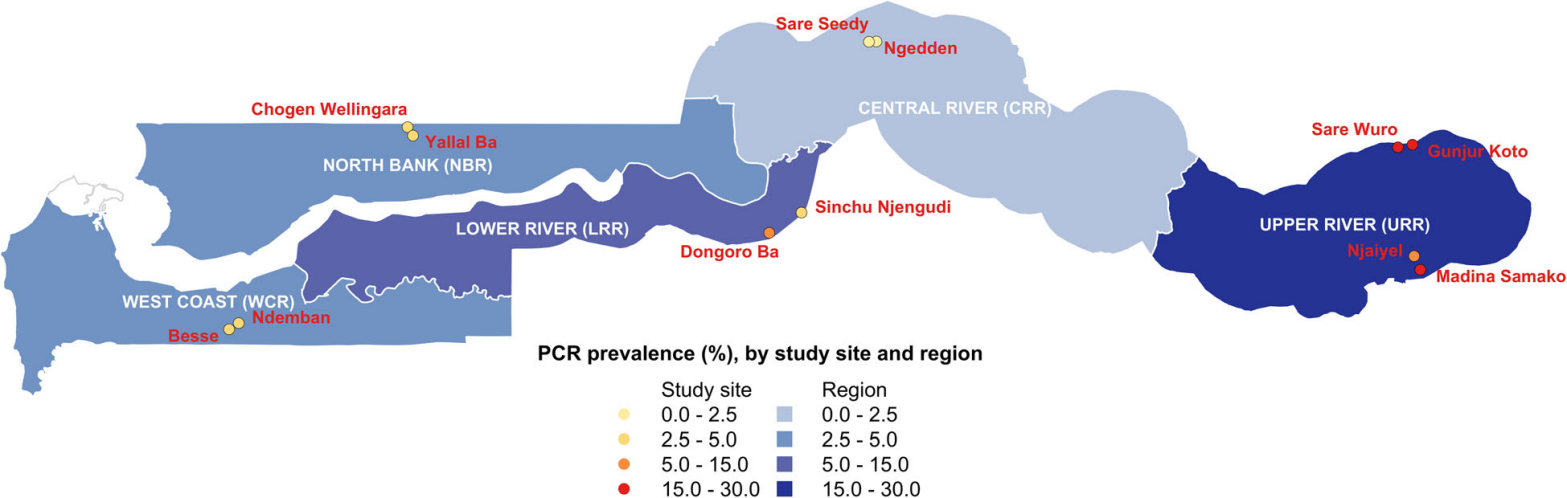
Map of Malaria Transmission Dynamics Study with regions and study villages by PCR prevalence

**Fig. 2:**
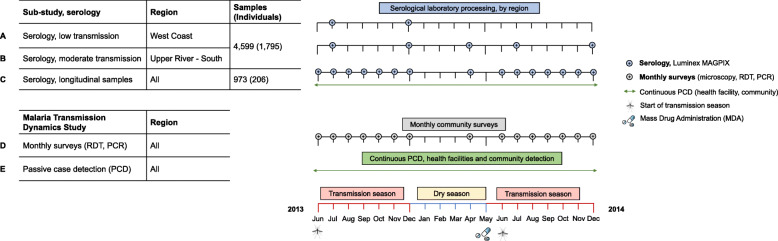
Study timelines. Malaria Transmission Dynamics Study timeline shown in black and green. Serological study timeline shown in blue for West Coast and Upper River Regions (low and moderate transmission settings, respectively). Serological analysis was conducted on samples from whole-village monthly surveys in N’demban and Besse in the West Coast Region (**a**), Njaiyal and Madina Samako in the Upper River Region (**b**), and longitudinal samples from individuals with a positive rapid diagnostic test (RDT) or polymerase chain reaction (PCR) test result during the Malaria Transmission Dynamics Study. Samples for serological analysis were processed on the Luminex MAGPIX; samples from monthly surveys were analysed using microscopy, rapid diagnostic tests (RDTs), and polymerase chain reaction (PCR)

To understand the dynamics of malaria infection and the impact of annual mass drug administration (MDA), a prospective cohort study was conducted from 2013 to 2015 in 12 villages across five administrative regions—West Coast (WCR), North Bank (NBR), Lower River (LRR), Central River (CRR), and Upper River (URR) Regions- as described by Mwesigwa et al. [13]. *Plasmodium falciparum* (*Pf*) prevalence measured by polymerase chain reaction (PCR) ranged from 2.27 to 19.60% in the Central River and Upper River Regions respectively (Fig. 1). Residents above 6 months of age were enrolled in the study, and monthly surveys were conducted during malaria transmission season from June to December each year, during the dry season in April 2014, and prior to the implementation of MDA in May and June 2014 and 2015 (Fig. 2). Individual finger prick blood samples were collected for haemoglobin estimation and on filter paper (Whatman 3 Corporation, Florham Park, NJ, USA) for molecular and serological analysis. Clinical malaria cases included individuals presenting with symptoms at health facilities (e.g. passive case detection) or individuals identified in villages by study nurses with history of fever in the previous 24 h or axillary temperature ≥ 37.5 °C and a positive rapid diagnostic test (RDT) result (Paracheck *Pf*, Orchid Biomedical System, India).

The serological study presented here is a subset of the Malaria Transmission Dynamics Study and included all available samples (*n* = 4599) from a selection of monthly surveys in four villages, totalling 1795 individuals (Fig. 2). In the West Coast Region (Besse and N’Demban), samples processed for serological analysis were from surveys conducted at the start of the transmission season in July 2013 (*N* = 534) and at the end of the season in December 2013 (*N* = 524). In the Upper River Region (URR), serological analysis included all samples collected in Njaiyal and Madina Samako in July 2013 (*N* = 778), December 2013 (*N* = 628), April (dry season) 2014 (*N* = 799), and December 2014 (*N* = 737) (Table 1). These regions represent extremes of two transmission intensities, with months selected at the start and end of the transmission season. Samples from clinical PCD cases were linked by study participant identification code to samples from the same individuals collected during routine monthly surveys. To further estimate the association between individual-level antibody responses and concurrent clinical or *Pf* infection, whole-village monthly survey samples in the West Coast Region and Upper River Region as described above were combined with an additional subset of 1244 longitudinal samples from 316 individuals who experienced a positive RDT or PCR test result or presented with clinical symptoms at any point during the Malaria Transmission Dynamics Study (Fig. 2). For these individuals, all available samples from the study were processed to longitudinally capture their serological responses before and after a positive RDT or PCR test result.

**Table 1:** Sample size and study subject characteristics by region and survey month. Sample size (*N*) reported as number of individuals in each village for each monthly survey and total number of compounds (with compound defined as a collection of households centrally located around a main residence). Study subject characteristics reported as number and percentage of individuals out of total individuals in each monthly survey. Dashed lines indicate survey months where serological analysis was not conducted in the West Coast Region

	July 2013	December 2013	April 2014	December 2014	
West Coast Region (WCR)					
Sample size (*N*)	**Individuals**				**Compounds**
*Besse*	400	387	–	–	69
*N*’*Demban*	134	137	–	–	24
Subtotal WCR	534	524	–	–	93
Gender, *n* (%)					
Male	240 (46.5)	226 (45.0)	–	–	
Female	276 (53.5)	276 (55.0)	–	–	
Age category, *n* (%)					
< 5 years	111 (21.4)	104 (20.6)	–	–	
5–15 years	186 (35.9)	194 (38.4)	–	–	
> 15 years	221 (42.7)	207 (41.0)	–	–	
LLIN use 24 h, *n* (%)	517 (96.8)	490 (94.2)	–	–	
Upper River Region (URR)					
Sample size (*N*)	**Individuals**				**Compounds**
*Njaiyal*	381	180	290	283	28
*Madina Samako*	397	448	509	454	43
Subtotal URR	778	628	799	737	71
Gender, *n* (%)					
Male	371 (47.9)	285 (45.7)	392 (49.4)	352 (47.9)	
Female	403 (52.1)	339 (54.3)	402 (50.6)	383 (52.1)	
Age category, *n* (%)					
< 5 years	164 (21.5)	143 (23.3)	183 (23.3)	169 (23.5)	
5–15 years	260 (34.1)	233 (37.9)	294 (37.5)	252 (35.0)	
> 15 years	338 (44.4)	239 (38.9)	308 (39.2)	299 (41.5)	
LLIN use 24 h, *n* (%)	278 (46.6)	244 (41.8)	278 (46.6)	294 (44.0)	

This study was approved by The Gambia Government/MRC Joint Ethics Committee (SCC1318). Verbal consent was first obtained at village sensitisation meetings, followed by individual written informed consent for all participants. Parents/guardians provided written consent for children less than 17 years, and assent was obtained from children between 12 and 17 years.

### Antigen selection and design

Antigens were selected from an initial screen of 856 candidates on an in vitro transcription and translation (IVTT) protein microarray based on correlation with malaria infection in children [11]. Antigens were generated and expressed in *Escherichia coli* (*E. coli*) as glutathione S-transferase (GST)-tagged fusion proteins [20–22], with the exception of *Pf*AMA1 expressed in *Pichia pastoris* as a histidine-tagged protein [15]. Protein purification was conducted by affinity chromatography (Glutathione Sepharose 4B, GE Healthcare Life Sciences) or HisPur Ni-NTA (Invitrogen) for GST- and His-tagged proteins, respectively, and concentration, quality, and purity of antigen yield assessed using a Bradford assay and SDS-PAGE. Bacterial lysate from culture of untransformed *E. coli* was used in assay buffers to eliminate background reactivity to *E. coli* proteins that were not specific to malaria target proteins.

Additionally, to account for potential non-malaria reactivity against GST-tagged fusion proteins, GST-coupled beads were included to quantify GST-specific immunoglobulin (IgG) responses and correct for non-specific binding. After laboratory processing, there were 71 participant samples with GST antibody responses above 1000 median fluorescence intensity (MFI), which was defined as the threshold to indicate potential non-malaria-specific binding, and were excluded from further analyses. Tetanus toxoid (TT, Massachusetts Biologic Laboratories) was also included as an internal positive control, assuming that vaccinated Gambians would show antibody responses to this protein target. A summary of antigen constructs and coupling conditions are detailed in Table 2.

**Table 2:** Summary of antigens in multiplex Luminex panel

Gene ID	Antigen name	Strain	Antigen bead coupling concentration (μg/mL)	Purification tag	Location	Description	Reference
PF3D7_0930300	*Pf*MSP1_19_	Wellcome	42.31	GST	Merozoite surface	19 kDa fragment of MSP1 molecule	[14]
PF3D7_1133400	*Pf*AMA1	FVO	3.90	His_x6_	Sporozoite/merozoite	Apical membrane antigen 1	[15]
PF3D7_1035300	*Pf*GLURP.R2	F32	9.22	N/A	Merozoite	Glutamate-rich protein R2	[16]
PF3D7_0731500	EBA175 RIII-V	3D7	408.32	GST	Merozoite	Erythrocyte-binding antigen-175 Region III-V	[17]
PF3D7_1335400	Rh2.2030	D10	244.30	GST	Merozoite	Reticulocyte binding protein homologue 2	[18]
PF3D7_0532100	Etramp5.Ag1	3D7	34.93	GST	iRBC/PVM	Early transcribed membrane protein 5	[19]
PF3D7_0402400	GEXP18	3D7	625.00	GST	Gametocytes	Gametocyte exported protein 18	[11]
PF3D7_0501100.1	HSP40.Ag1	3D7	42.54	GST	iRBC/gametocytes	Heat shock protein 40, type II	[11]
–	GST	–	85.99	–	–	GST expression tag	
–	TT	–	61.52	–	–	Tetanus toxoid	

*iRBC* infected red blood cell, *PVM* parasitophorous vacuole membrane, *GST* glutathione S-transferase

### Laboratory procedures

Antigen-specific antibody responses were quantified using the Luminex MAGPIX protocol described in Wu et al. 2019 [23]. Plasma was eluted from 6 mm dried blood spots (DBS) (4 μl whole blood equivalent) and shaken overnight at room temperature in 200 μl of protein elution buffer containing phosphate buffered saline (PBS) (pH 7.2), 0.05% sodium azide, and 0.05% Tween-20, yielding an initial 1:50 sample dilution. One day prior to assay processing, samples were diluted to a final 1:500 dilution using 10 μl of the 1:50 pre-dilution sample and 90 μl of blocking Buffer B to prevent non-specific binding (1xPBS, 0.05% Tween, 0.5% bovine serum albumin (BSA), 0.02% sodium azide, 0.1% casein, 0.5% polyvinyl alcohol (PVA), 0.5% polyvinyl pyrrolidone (PVP), and 1500 μg/ml *E. coli* extract). Negative and positive controls were also incubated 1 day prior in Buffer B, with negative controls prepared at a 1:500 dilution and Gambian pooled positive controls in a 6-point 5-fold serial dilution (1:10–1:31,250). The positive control was based on a pool of 22 serum samples from malaria hyper-immune individuals in The Gambia, and ten individual plasma samples from European malaria-naive adults were used as negative controls.

Samples were prepared for diagnostic PCR as described by Mwesigwa et al. [13]. Briefly, DNA was extracted from three 6-mm DBS using the automated QIAxtractor robot (Qiagen). Negative and positive (3D7) controls were included to control for cross contamination and DNA extraction efficiency, respectively. The DBS were lysed by incubation in tissue digest buffer at 60 °C for 1 h and digested eluates were applied onto capture plates, washed, and the DNA eluted into 80 μl. The extracted DNA (4 μl) was used in a nested PCR, amplifying the multi-copy *Plasmodium* ribosomal RNA gene sequences using genus and species specific primers [24]. All PCR products were run using the QIAxcel capillary electrophoresis system (Qiagen), using the screening cartridge and 15–1000 bp-alignment marker. Results were exported and double scored using both the QIAxcel binary scoring function and manually by visualisation of the gel images and discrepancies were scored by a third independent reader. All readers were blinded to participant survey data.

### Statistical analyses

Data analysis was based on total IgG levels to five antigens as potential markers of sero-incidence [11]—early transcribed membrane protein 5 (Etramp5.Ag1), gametocyte export protein 18 (GEXP18), heat shock protein 40 (HSP40.Ag1), erythrocyte-binding antigen 175 RIII-V (EBA175), and reticulocyte binding protein homologue 2 (Rh2.2030). Three antigens associated with long-lived antibody response—*P. falciparum* merozoite surface antigen 1 19-kDa carboxy-terminal region (*Pf*MSP1_19_), *P. falciparum* apical membrane antigen 1 (*Pf*AMA1), and *P. falciparum* glutamate-rich protein, region 2 (*Pf*GLURP.R2)—were included as a comparison, which have historically been used to assess sero-conversion rates over time [8, 9] and have also previously been studied in The Gambia [6, 7]. For antigens associated with long-lived antibodies (*Pf*MSP1_19_, *Pf*AMA1, *Pf*GLURP.R2), individuals residing in an endemic region previously exposed to malaria, but not recently infected, may still have residual antibody levels that are significantly higher than malaria-naïve individuals in non-endemic regions. Therefore, a two-component Gaussian mixture model was used to define distributions of negative and positive antibody levels, expressed in units of median fluorescence intensity (MFI). Sero-positivity thresholds were defined as the mean log MFI values plus two standards deviations of the negative distribution [25]. Mixture models were estimated using the ‘*normalmixEM*’ function in the ‘*mixtools*’ package v1.0.4 in R version 3.6.1. For antigens associated with shorter-lived antibodies where statistical evidence of a bimodal distribution of antibody responses in the population was not strong given the more rapid decay of antibody levels post-infection—Etramp5.Ag1, GEXP18, HSP40.Ag1, EBA175, and Rh2.2030—the sero-positivity threshold was defined by the mean log MFI plus three standard deviations of 71 malaria-naïve European blood donors used as negative controls.

Individual-level association between antibody response and the concurrent odds of clinical malaria (passively detected via the health facility or study nurses in the community) or asymptomatic *P. falciparum* infection (actively detected using PCR from monthly survey samples) were assessed using generalised estimating equations (GEE). Analysis was adjusted for age group (1–5 years, 6–15 years, and greater than 15 years) and use of long-lasting insecticide-treated nets (LLINs) in the last 24 h and allowed for clustering at the compound level, where compound is a collection of households centrally located around a main residence. The magnitude of the association between antibody response and odds of infection was evaluated for interaction with age group. Based on a subset of longitudinal samples, individual-level association between antibody response and recent infection in the previous 4 months (as opposed to current infection at the same time point) was also assessed using a GEE model, adjusted for age group, LLIN use, and random effects at the compound level, as above.

Using a GEE model, individual-level odds of clinical malaria or asymptomatic infection was assessed for association with residing in the same compound as a sero-positive individual. Similarly, the association between individual odds of infection and compound-level sero-prevalence (< 50% or > 50%) was assessed using a mixed-effects generalised linear model adjusted for age group and LLIN use and random effects at the compound level, which also accounts for the potential effect of geographical differences in transmission intensity. To ensure that estimates are not biased by the sero-prevalence of small compounds, analysis was weighted by the number of individuals in the compound (whose sero-status was assessed) and only included compounds with at least four individuals. Models were fit using the ‘geeM’ and ‘lme4’ packages in R version 3.14.

Village-level sero-prevalence amongst children aged 2–10 years in the West Coast Region and Upper River Region in July and December 2013 (*n* = 1001) was compared against all-age clinical and *P. falciparum* infection incidence rates from the same months, the latter of which were previously reported by Mwesigwa et al. [13]. Monthly clinical and *P. falciparum* infection incidence rates were defined respectively as the number of new clinical cases or *P. falciparum* infections (PCR-positive individuals who were PCR-negative in the previous monthly survey) divided by total person years at risk (PYAR). This age range was selected to align with other routine surveillance metrics, such as annual parasite index (API), commonly using this age group as a sentinel population. The strength of the relationship between village-level incidence rates and sero-prevalence for each antigen was assessed using Pearson’s correlation coefficient. Data from April and December 2014 were not included in the analysis because clinical incidence and *Pf* infection rates from these surveys have not yet been reported.

## Results

### Village-level sero-prevalence

Data from 2001 individuals were available for analysis of antibody responses from 5572 samples between June 2013 and December 2014 (Fig. 2). Based on whole-village monthly survey data in the West Coast and Upper River Regions, slightly more than half of participants were female in both the West Coast Region (55.0%) and the Upper River Region (54.3%), and there was higher LLIN use in the West Coast Region (96.8%) compared to the Upper River Region (46.6%) (Table 1). In the West Coast Region, *P. falciparum* infection rate in December 2013 (0.67 95% CI 0.40–1.13) was three times higher than at the start of the transmission season in July 2013 (0.23 95% CI 0.13–0.39) (Table 3). In the Upper River Region, *P. falciparum* infection rate in December 2013 (2.87 95% CI 2.36–3.50) was five times higher than July 2013 (0.56 95% CI 0.42–0.74) (Table 3). Sero-prevalence amongst 2–10 years olds in the West Coast Region ranged from 1.0% (95% CI 0.0–2.3) for Rh2.2030 in July 2013 (start of the transmission season) to 13.7% (95% CI 8.9–18.5) for Etramp5.Ag1 in December 2013 (end of transmission season) (Table 3). In the Upper River Region, sero-prevalence across all antigens was higher, ranging from 5.1% (95% CI 2.5–7.7) for *Pf*MSP1_19_ in July 2013 to 36.3% (95% CI 30.2–42.3) for GEXP18 in December 2013 (Table 3).

**Fig. 3:**
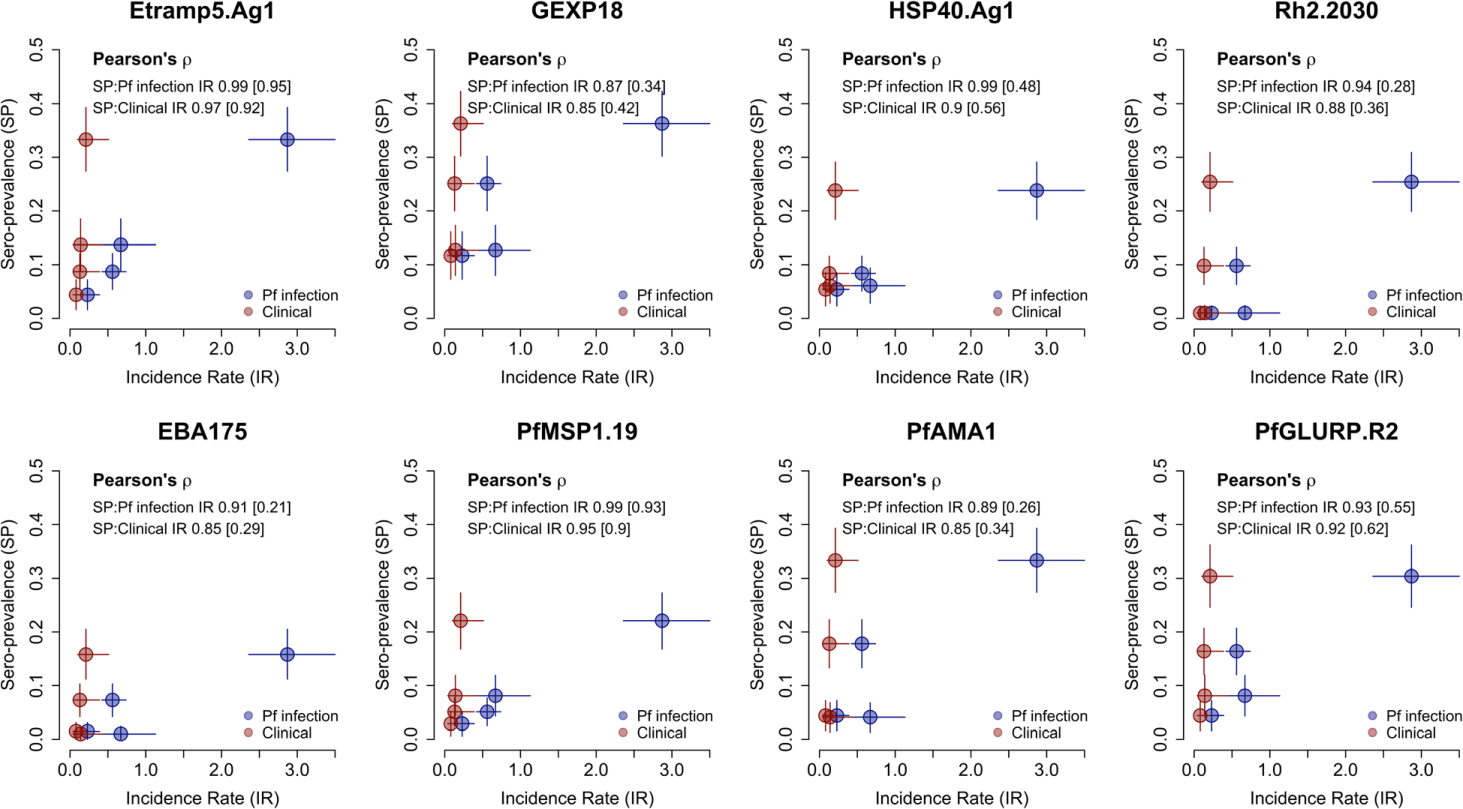
Pearson’s correlation between regional sero-prevalence (ages 2–10 years) and all-age incidence rates for clinical malaria and *Pf* infection. Correlations between sero-prevalence and incidence rates for clinical malaria (new clinical episodes divided by person-years at risk) in red and *Pf* infection (new PCR infections divided by person-years at risk) in blue were based on data from the West Coast Region (WCR) and the Upper River Region (URR) in July and December 2013. Correlations excluding data from URR December 2013 shown in brackets

In the Upper River Region in 2014, no statistically significant differences in sero-prevalence amongst 2–10 year olds were observed for any antigen (Additional file 1 – Table S1). It should be noted that MDA was implemented in May–June 2014, so results may not be comparable to 2013 prior to intervention. Sero-prevalence in this age group was highly correlated with clinical malaria and *P. falciparum* infection incidence rates (Fig. 3, Table 4).

**Table 3:** Regional sero-prevalence (ages 2–10 years) by antigen and all-age incidence rates for clinical malaria and *Pf* infection. Mean sero-prevalence (%) and incidence rates with 95% CI shown in parentheses

	West Coast Region (WCR)		Upper River Region (URR)	
	July 2013	December 2013	July 2013	December 2013
Clinical incidence rate	0.08 (0.04–0.21)	0.14 (0.04–0.44)	0.13 (0.04–0.39)	0.21 (0.1–0.51)
*Pf* infection rate	0.23 (0.13–0.39)	0.67 (0.40–1.13)	0.56 (0.42–0.74)	2.87 (2.36–3.50)
Sero-prevalence (ages 2–10 years)				
	*n* = 205	*n* = 197	*n* = 275	*n* = 324
Etramp5.Ag1	4.4% (1.6–7.2)	13.7% (8.9–18.5)	8.7% (5.4–12.1)	33.3% (27.4–39.3)
GEXP18	11.7% (7.3–16.1)	12.7% (8.0–17.3)	25.1% (20.0–30.2)	36.3% (30.2–42.3)
HSP40.Ag1	5.4% (2.3–8.5)	6.1% (2.8–9.4)	8.4% (5.1–11.6)	23.8% (18.4–29.1)
Rh2.2030	1.0% (0.0–2.3)	1.0% (0.0–2.4)	9.8% (6.3–13.3)	25.4% (19.9–30.9)
EBA175	1.5% (0.0–3.1)	1.0% (0.0–2.4)	7.3% (4.2–10.3)	15.8% (11.2–20.5)
*Pf*MSP1_19_	2.9% (0.6–5.2)	8.1% (4.3–11.9)	5.1% (2.5–7.7)	22.1% (16.8–27.3)
*Pf*AMA1	4.4% (1.6–7.2)	4.1% (1.3–6.8)	17.8% (13.3–22.3)	33.3% (27.4–39.3)
*Pf*GLURP.R2	4.4% (1.6–7.2)	8.1% (4.3–11.9)	16.4% (12.0–20.7)	30.4% (24.6–36.2)

**Table 4:** Pearson’s correlation coefficients between regional sero-prevalence (ages 2–10 years) and all-age incidence rates for clinical malaria and *Pf* infection. Correlation estimates are based on data from the West Coast Region (WCR) and Upper River Region (URR) in July and December 2013. Correlations excluding data from URR December 2013 are shown in brackets

Antigen	Pearson’s correlation coefficient	
	Sero-prevalence vs. clinical incidence rate	Sero-prevalence vs. ***Pf*** infection incidence rate
Etramp5.Ag1	0.97 [0.92]	0.99 [0.95]
GEXP18	0.85 [0.42]	0.87 [0.34]
HSP40.Ag1	0.90 [0.56]	0.99 [0.48]
Rh2.2030	0.88 [0.36]	0.94 [0.28]
EBA175	0.85 [0.29]	0.91 [0.21]
*Pf*MSP1_19_	0.95 [0.90]	0.99 [0.93]
*Pf*AMA1	0.85 [0.34]	0.89 [0.26]
*Pf*GLURP.R2	0.92 [0.62]	0.93 [0.55]

In both regions in July and December 2013, correlations between *P. falciparum* infection rate and sero-prevalence were strongest for Etramp5.Ag1 (*ρ* = 0.99), HSP40.Ag1 (*ρ* = 0.99), *Pf*MSP1_19_ (*ρ* = 0.99), and Rh2.2030 (*ρ* = 0.94). Incidence of clinical malaria had the strongest correlations with Etramp5.Ag1 (*ρ* = 0.97), *Pf*MSP1_19_ (*ρ* = 0.95), *Pf*GLURP.R2 (*ρ* = 0.92), and HSP40.Ag1 (*ρ* = 0.90). After excluding December 2013 data from the Upper River Region as a potential outlier, correlations with both clinical incidence and *P*. *falciparum* infection rates remained high for Etramp5.Ag1 (*p* = 0.92 and *p* = 0.95, respectively) and P*f*MSP1_19_ (*p* = 0.90 and *p* = 0.93, respectively). These correlations are based on few data points, with only three observations when excluding the Upper River Region in December 2013.

### Individual-level association between clinical malaria, asymptomatic *P*. *falciparum* infection, and concurrent sero-positivity

**Fig. 4:**
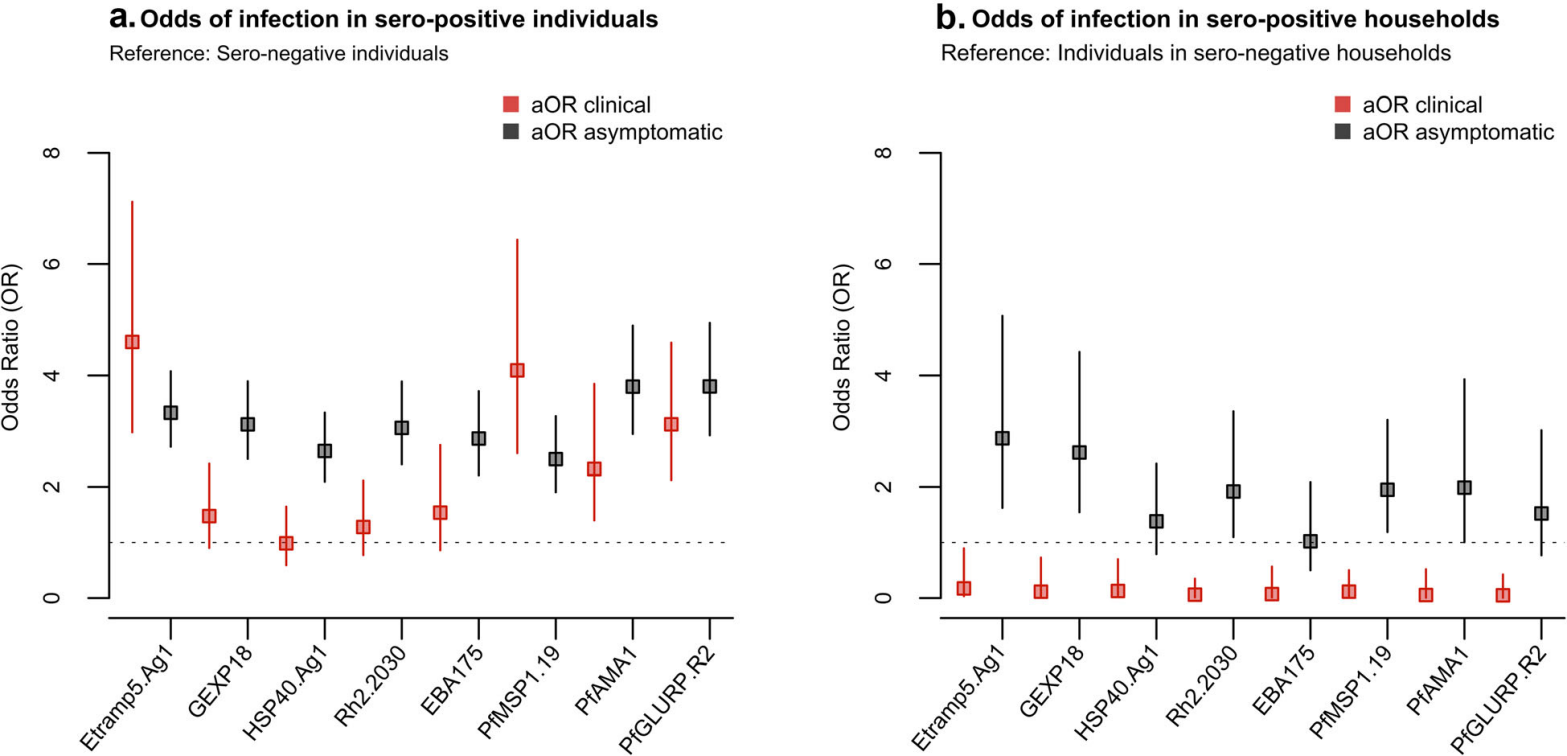
Odds clinical malaria and asymptomatic infection by **a** individual sero-positivity status **b** residing in the same compound as a sero-positive individual. Analyses is adjusted for age group (< 5 years, 5–15 years, and > 15 years) and LLIN use in the last 24 h and weighted by compound size

The odds of clinical malaria were higher in individuals sero-positive to six (out of eight) of the antigens analysed (Etramp5.Ag1, GEXP18, Rh2.2030, *Pf*MSP1_19_, *Pf*AMA1, and *Pf*GLURP.R2) (Fig. 4a, Table 5). After adjusting for age and LLIN use, clinical malaria was associated with concurrent sero-positivity to four antigens (Etramp5.Ag1, *Pf*MSP1_19,_ *Pf*AMA1, and *Pf*GLURP.R2), while odds of asymptomatic infection were strongly associated with sero-positivity to all antigens. Amongst individuals sero-positive for Etramp5.Ag1, there was increased odds of both clinical malaria (aOR 4.60, 95% CI 2.98–7.12, *p* < 0.001) and asymptomatic infection (aOR 3.33, 95% CI 2.72–4.08, *p* < 0.001) (Table 5). Association with clinical malaria was slightly lower in individuals sero-positive for *Pf*MSP1_19_ (aOR 4.09, 95% CI 2.60–6.44, *p* < 0.001), *Pf*GLURP.R2 (aOR 3.12, 95% CI 2.12–4.59, *p* < 0.001), and *Pf*AMA1 (aOR 2.32, 95% CI 1.40–3.85 *p* = 0.001). Odds of asymptomatic infection in individuals sero-positive to GEXP18 (aOR 3.12, 95% CI 2.50–3.90, *p* < 0.001), Rh2.2030 (aOR 3.06, 95% CI 2.40–3.89, *p* < 0.001), *Pf*AMA1 (aOR 3.80, 95% CI 2.95–4.90, *p* < 0.001), and *Pf*GLURP.R2 (aOR 3.80 95% CI 2.92–4.95, *p* < 0.001) was similar to Etramp5.Ag1. Additionally, a subset of longitudinal samples were used to explore the dynamics of antibody responses following infection. After adjusting for age group and LLIN use, antibody responses to six out of eight antigens were significantly associated with infection (clinical, RDT-positive or PCR-positive) in the previous 4 months. Antibody levels against Etramp5.Ag1 were most strongly associated with previous infection (aOR 1.44 95% CI 1.19–1.75, *p* < 0.001), followed by *Pf*AMA1 (aOR 1.33 95% CI 1.16–1.53, *p* < 0.001), EBA175 (aOR 1.26 95% CI 1.09–1.46, *p* = 0.002), GEXP18 (aOR 1.23 95% CI 1.03–1.47, *p* = 0.021), *Pf*MSP1_19_ (aOR 1.21 95% CI 1.04–1.41, *p* = 0.012), and Rh2.2030 (aOR 1.20 95% CI 1.03–1.39, *p* = 0.020).

**Table 5:** Odds of clinical malaria and asymptomatic infection by individual-level serological status, unadjusted and adjusted for age group (< 5 years, 5–15 years, and > 15 years) and LLIN use in the last 24 h

Odds of clinical malaria or asymptomatic infection amongst sero-positive individuals, by antigen						
	Unadjusted			Adjusted		
	OR	95% CI	***p*** value	aOR	95% CI	***p*** value
Etramp5.Ag1						
*Clinical malaria*	5.88	3.44–10.03	< 0.001	4.60	2.98–7.12	< 0.001
*Asymptomatic infection*	3.20	2.64–3.88	< 0.001	3.33	2.72–4.08	< 0.001
GEXP18						
*Clinical malaria*	2.57	1.44–4.61	0.002	1.48	0.90–2.41	0.122
*Asymptomatic infection*	2.94	2.38–3.64	< 0.001	3.12	2.50–3.90	< 0.001
HSP40.Ag1						
*Clinical malaria*	1.67	0.88–3.18	0.119	0.99	0.59–1.65	0.956
*Asymptomatic infection*	2.53	2.06–3.10	< 0.001	2.64	2.09–3.33	< 0.001
Rh2.2030						
*Clinical malaria*	2.20	1.10–4.38	0.025	1.28	0.77–2.12	0.338
*Asymptomatic infection*	2.45	1.98–3.03	< 0.001	3.06	2.40–3.89	< 0.001
EBA175						
*Clinical malaria*	1.37	0.67–2.81	0.390	1.54	0.86–2.75	0.147
*Asymptomatic infection*	2.09	1.74–2.51	< 0.001	2.86	2.21–3.72	< 0.001
*Pf*MSP1_19_						
*Clinical malaria*	3.83	1.95–7.51	< 0.001	4.09	2.60–6.44	< 0.001
*Asymptomatic infection*	2.29	1.82–2.88	< 0.001	2.49	1.90–3.27	< 0.001
*Pf*AMA1						
*Clinical malaria*	2.21	1.25–3.90	0.006	2.32	1.40–3.85	0.001
*Asymptomatic infection*	2.62	2.12–3.24	< 0.001	3.80	2.95–4.90	< 0.001
*Pf*GLURP.R2						
*Clinical malaria*	2.17	1.28–3.68	0.004	3.12	2.12–4.59	< 0.001
*Asymptomatic infection*	2.38	1.96–2.89	< 0.001	3.80	2.92–4.95	< 0.001

### Individual-level odds of clinical malaria, asymptomatic infection when residing in compounds with sero-positive individuals

The odds of clinical malaria were reduced for individuals residing in the same compound with an individual sero-positive to any of the eight antigens (Fig. 4b, Table 6), from an 83% reduction if residing in the same compound as an individual sero-positive for Etramp5.Ag1 (aOR 0.17, 95% CI 0.03–0.89, *p* = 0.036) to a 95% reduction if residing in the same compound as someone sero-positive for *Pf*AMA1 (aOR 0.05, 95% CI 0.01–0.52, *p* = 0.011). Conversely, the odds of asymptomatic infection were positively associated with residing in the same compound as sero-positive individuals for five antigens (Etramp5.Ag1, GEXP18, Rh2.2030, *Pf*MSP1_19,_ and *Pf*AMA1). Adjusted odds of asymptomatic infection was nearly 3-fold higher if residing with an individual sero-positive for Etramp5.Ag1 (aOR 2.87, 95% CI 1.62–5.07, *p* < 0.001) and for GEXP18 (aOR 2.61, 95% CI 1.54–4.42, *p* < 0.001). If residing in a compound with individuals sero-positive to Rh2.2030, *Pf*MSP1_19_, or *Pf*AMA1, the odds of asymptomatic infection were all nearly twice as high as individuals living in compounds sero-negative to these antigens (Table 6).

**Table 6:** Odds of clinical malaria and asymptomatic *Pf* infection by compound serological status. Estimates are weighted by compound size and shown as unadjusted odds ratios (OR) and adjusted odds ratios (aOR) for age group (< 5 years, 5–15 years, and > 15 years)

Odds of clinical malaria or asymptomatic *Pf* infection for individuals residing in sero-positive compounds						
	Unadjusted			Adjusted		
	OR	95% CI	***p*** value	aOR	95% CI	***p*** value
Etramp5.Ag1						
*Clinical malaria*	0.19	0.03–1.06	0.059	0.17	0.03–0.89	0.036
*Asymptomatic infection*	2.83	1.62–4.96	< 0.001	2.87	1.62–5.07	< 0.001
GEXP18						
*Clinical malaria*	0.13	0.02–0.90	0.039	0.11	0.02–0.73	0.022
*Asymptomatic infection*	2.65	1.58–4.47	< 0.001	2.61	1.54–4.42	< 0.001
HSP40.Ag1						
*Clinical malaria*	0.14	0.02–0.80	0.027	0.13	0.02–0.70	0.018
*Asymptomatic infection*	1.32	0.77–2.28	0.316	1.38	0.79–2.41	0.257
Rh2.2030						
*Clinical malaria*	0.07	0.01–0.48	0.006	0.06	0.01–0.35	0.002
*Asymptomatic infection*	1.69	0.96–2.99	0.070	1.92	1.10–3.36	0.022
EBA175						
*Clinical malaria*	0.07	0.01–0.61	0.016	0.07	0.01–0.57	0.013
*Asymptomatic infection*	1.00	0.50–2.01	0.992	1.02	0.50–2.09	0.954
*Pf*MSP1_19_						
*Clinical malaria*	0.15	0.03–0.87	0.034	0.12	0.03–0.50	0.004
*Asymptomatic infection*	1.87	1.15–3.06	0.012	1.95	1.19–3.20	0.008
*Pf*AMA1						
*Clinical malaria*	0.06	0.01–0.62	0.018	0.05	0.01–0.52	0.011
*Asymptomatic infection*	1.96	1.01–3.81	0.046	1.99	1.00–3.93	0.048
*Pf*GLURP.R2						
*Clinical malaria*	0.06	0.01–0.53	0.012	0.05	0.01–0.43	0.006
*Asymptomatic infection*	1.59	0.85–2.97	0.145	1.52	0.77–3.01	0.228

The prevalence of sero-positive compounds for long-lived antibody responses varied by antigen and region but remained generally stable throughout the transmission season. For *Pf*MSP1_19_, prevalence of sero-positive compounds was 32.2% (95% CI 14.9–49.5) in July 2013 and 39.8% (95% CI 23.6–56.0) in December 2013, while prevalence of compounds sero-positive for *Pf*AMA1 and *Pf*GLURP.R2 ranged between 77.0% (95% CI 66.9–87.1) in July 2013 and 80.7% (95% CI 71.5–89.9) in December 2013 (Additional file 1 – Table S1). In the Upper River Region, nearly all compounds were sero-positive to *Pf*AMA1 and *Pf*GLURP.R2, from 96.3% (95% CI 91.2–100) in July 2013 to 100% in December 2014, but prevalence was slightly lower for *Pf*MSP1_19,_ which was between 70.4% (95% CI 55.9–84.9) in July 2013 and 88.6% (95% CI 80.7–96.5) in December 2014. By contrast, prevalence of sero-positive compounds for short-lived antibody responses were lower in both regions, ranging from 41.4% (95% CI 25.3–57.5) for Etramp5.Ag1 to 63.2% (95% CI 50.5–76.0) for EBA175 in July 2013 in the West Coast Region and from 72.2% (95% CI 58.2–86.3) for Etramp5.Ag1 in July 2013 to 97.1% (95% CI 93.2–100) for GEXP18 in December 2014 in the Upper River Region.

### Association between individual-level clinical malaria, asymptomatic infection, and compound sero-prevalence

**Fig. 5:**
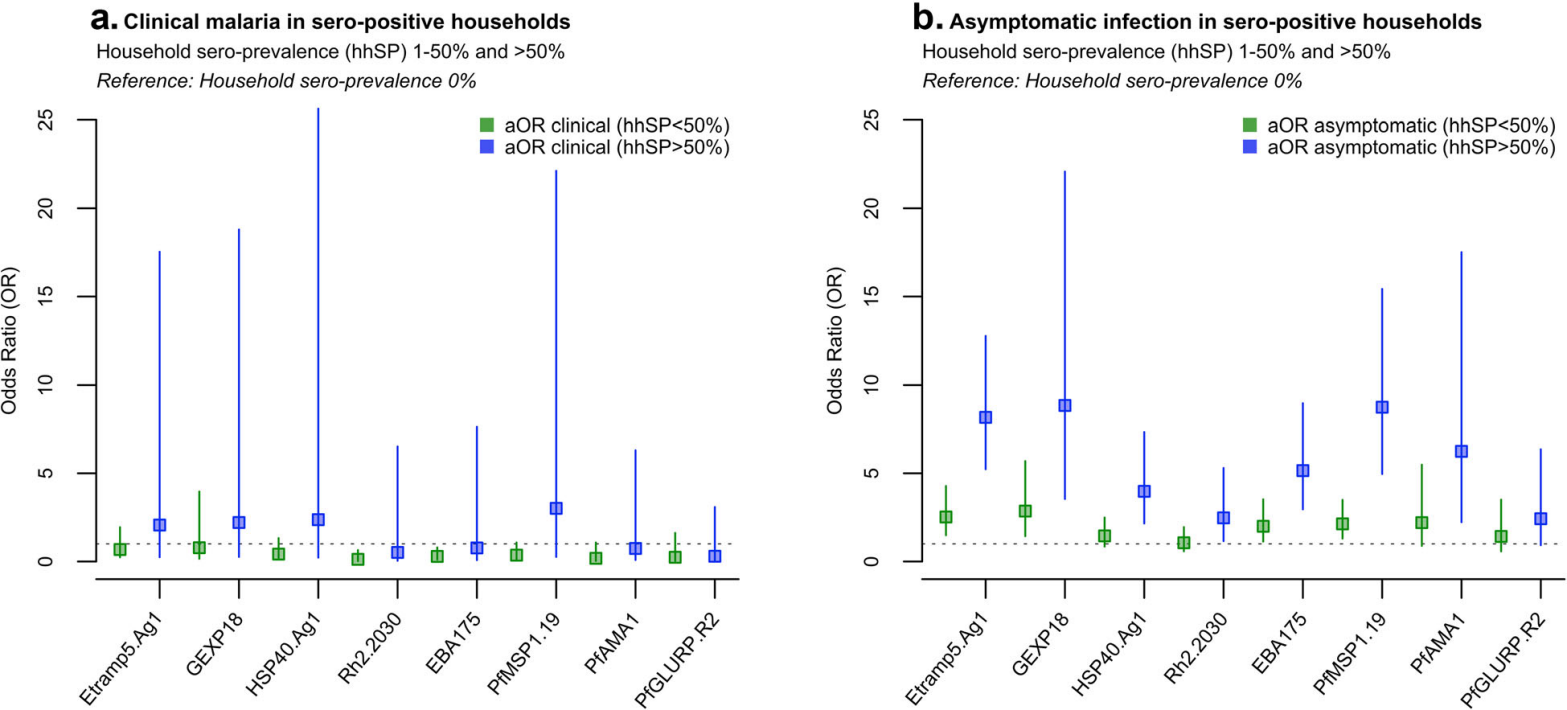
Odds of clinical malaria by compound sero-prevalence (**a**) and odds of asymptomatic infection by compound sero-prevalence (**b**). Analyses is adjusted for age group (< 5 years, 5–15 years, and > 15 years) and LLIN use in the last 24 h and weighted by compound size

When stratifying by compound-level sero-prevalence, mean regression estimates found a reduced odds of clinical malaria if residing in a compound with sero-prevalence less than 50% for all antigens and an increased odds of clinical malaria in compounds with greater than 50% sero-prevalence for four antigens (Etramp5.Ag1, GEXP18, HSP40.Ag1, and *Pf*MSP1_19_) (Fig. 5a, Additional file 2 - Tables S3, S7, S11, S19, and S23). However, these results were not statistically significant. There was strong statistical evidence for increasing odds of asymptomatic infection with increasing compound sero-prevalence for two antigens (Etramp5.Ag1 and *Pf*MSP1_19_) (Fig. 5b, Additional file 2 - Tables S4, S24) and weaker statistical evidence for increasing odds of asymptomatic infection for HSP40.Ag1 and Rh2.2030 (Additional file 2 - Tables S14, S16). Average compound size in regions with less than 15% PCR prevalence (West Coast, North Bank, Lower River and Central River Regions) was 9.3 (95% CI 7.9–10.7) individuals, and in regions with more than 15% PCR prevalence (Upper River Regions South and North), average compound size was slightly larger (16.3 95% CI 13.4–19.1 individuals) (Additional file 1 – Figure S1). Across all compounds, the average age range within a household was 54.2 years (95% CI 51.2–57.1), with an average minimum age of 2.6 years (95% CI 2.1–3.0) and a maximum age of 56.7 years (95% CI 53.8–59.8) (Additional file 1 – Figure S1).

Antigens most associated with increased odds of asymptomatic infection were Etramp5.Ag1, GEXP18, and *Pf*MSP1_19_. For Etramp5.Ag1, aOR ranged from 2.52 (95% CI 1.49–4.28, *p* = 0.001) in compounds with less than 50% sero-prevalence to aOR 8.17 (95% CI 5.23–12.76, *p* < 0.001) in compounds with sero-prevalence greater than 50%, compared to sero-negative compounds as the baseline reference (Additional file 2 - Table S4). For GEXP18, aOR ranged from 2.86 (95% CI 1.44–5.69, *p* = 0.003) to 8.85 (95% CI 3.54–22.08, *p* < 0.001) (Additional file 2 - Table S8). For *Pf*MSP1_19_, aOR ranged from 2.13 (95% CI 1.30–3.50, *p* = 0.003) to aOR 8.75 (95% CI 4.96–15.43, *p* < 0.001) (Additional file 2 - Table S24). For HSP40.Ag1, EBA175, and *Pf*AMA1, statistically strong evidence for increased odds of asymptomatic infection was only observed in compounds with greater than 50% sero-prevalence (Additional file 2 - Tables S12, S20, S28).

**Fig. 6:**
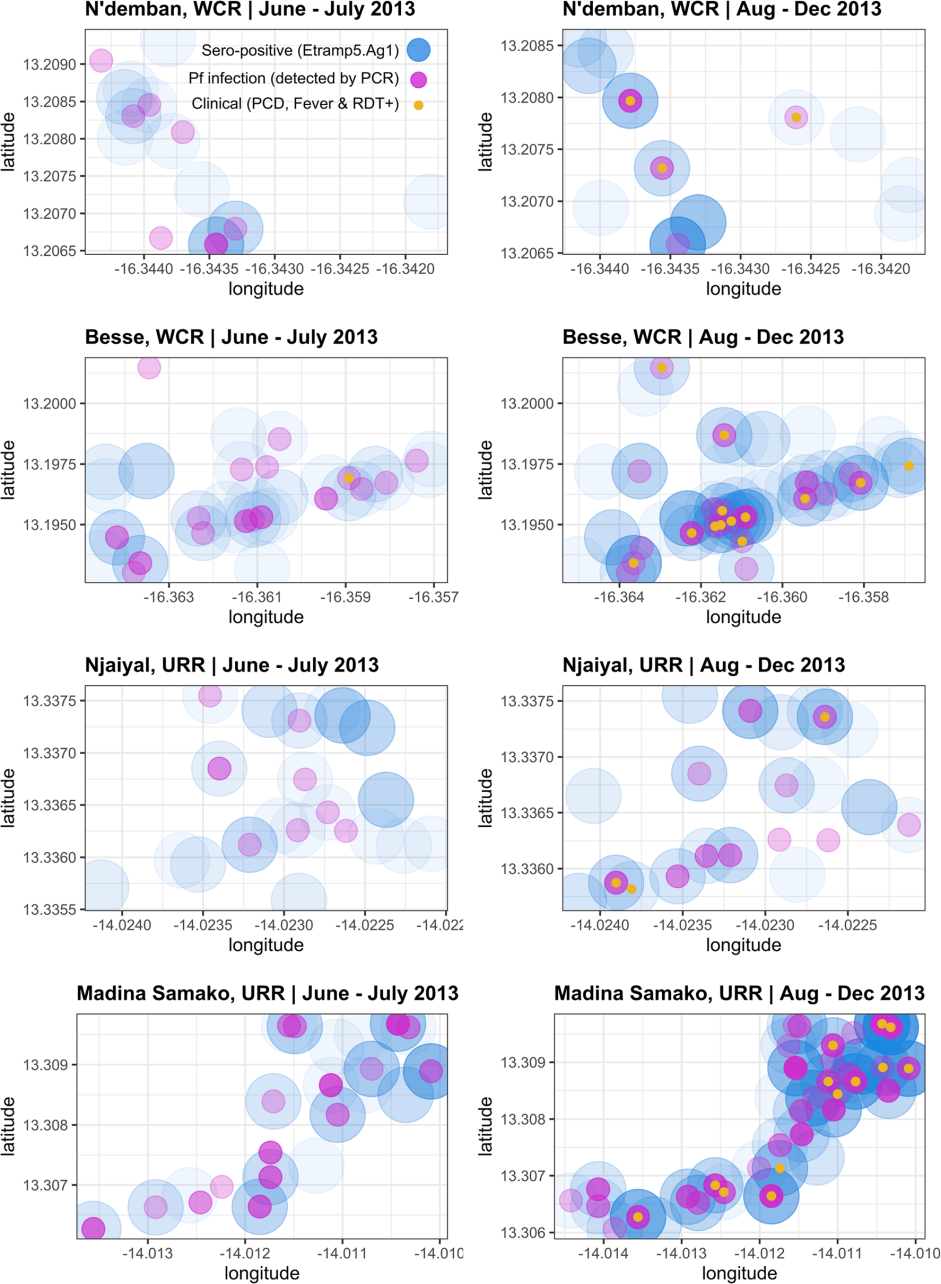
Household geolocation of Etramp5.Ag1 sero-positive individuals, clinical malaria, and *Pf* infections across four villages. Spatial distribution of infections shown for lower transmission (N’demban) and higher transmission (Besse) villages in the West Coast Region (WCR) and lower transmission (Njaiyal) and higher transmission (Madina Samako) villages in the Upper River Region (URR). Infections at the start of the wet season (June–July 2013) shown on the left and during the wet and transmission season (August–December 2013) on the right

The spatial distribution of infections at both the start and end of the transmission season (June–July 2013 vs. August–December 2013) suggests that clinical malaria and asymptomatic infection is localised near households with Etramp5.Ag1 sero-positive individuals (Fig. 6). There are a number of Etramp5.Ag1 sero-positive households at both the start and during the wet season without concurrent clinical or asymptomatic infections present. However, this is more pronounced for *Pf*AMA1, where sero-positivity could reflect a longer history of exposure than Etramp5.Ag1 (Additional file 3 – Figure S1). While there are a limited number of clinical cases between June and July 2013, from August to December 2013, these cases occur in proximity to households with asymptomatic and Etramp5.Ag1 sero-positive individuals (Fig. 6).

## Discussion

We investigated the association between antibody responses and clinical and parasitological endpoints at the individual, household, and population level across The Gambia. At the individual level, clinical malaria was highly correlated with concurrent sero-positivity to Etramp5.Ag1, *Pf*MSP1_19_, *Pf*AMA1, and *Pf*GLURP.R2, while asymptomatic infection was correlated with sero-positivity to all antigens included in this study. The strongest associations were between asymptomatic infection and sero-positivity to Etramp5.Ag1 and *Pf*AMA1. Village-level sero-prevalence amongst children 2–10 years against antigens Etramp5.Ag1, HSP40.Ag1, and *Pf*MSP1_19_ showed the highest correlations with clinical incidence and *Pf* infection rates. In addition to current infection, antibody responses to several antigens, particularly Etramp5.Ag1, were associated with prior infection in the last 4 months, highlighting the potential for cross-sectional serological surveillance to identify areas of recent malaria exposure, even after parasites have been cleared and are no longer detectable by PCR. To better inform how these antigenic targets can be used to estimate time since infection, future research is needed to quantify the half-life of antibody responses and determine the sensitivity and specificity for identifying previous infection across varying epidemiological settings. This will require more detailed cohort studies with intensive follow-up for extended periods (ideally more than 1 year) and with sufficient sample sizes to capture individual heterogeneity [26].

At the household level, individuals had increased odds of asymptomatic infection when residing in a compound with greater than 50% sero-prevalence to all antigens, with the highest odds associated with sero-prevalence to Etramp5.Ag1, GEXP18, and *Pf*MSP1_19_. While these results cannot confirm a causal relationship between serological responses and clinical outcomes or asymptomatic infection, they indicate that infected individuals tend to be concurrently sero-positive to most of the serological markers assessed. There was strong evidence of reduced odds of clinical malaria for individuals residing in the same compound as an individual sero-positive to any of the antigens, suggesting a protective effect. In fact, previous studies suggest that several of the antigens investigated in this study—EBA175, Rh2, *Pf*GLURP.R2, *Pf*AMA1, and *Pf*MSP1—have a functional role in mediating protective immunity if antibodies are acquired at a sufficiently high magnitude [27–29].

While *Pf*MSP1_19_ and *Pf*AMA1 have been used extensively to measure medium- and long-term trends in transmission intensity, these markers may be less sensitive to changes in transmission at higher endemicities or to short-term fluctuations due to the rapid acquisition of antibodies and the long half-life of sero-positivity for some blood-stage antigens [30–33]. Individuals with clinical malaria or with long-lived antibody responses in low transmission regions of western Gambia could indicate foci of malaria transmission that have been ongoing for several transmission seasons or years. In areas where parasite density is low and often missed by RDTs and microscopy, using serological markers could support targeted interventions to clear or eliminate these residual reservoirs of human infection. Antigens in this study associated with shorter-lived antibodies—Etramp5.Ag1, GEXP18, and HSP40.Ag1—were positively correlated with clinical malaria and asymptomatic infection. Antibody responses to these antigens have been shown to decrease rapidly after infection in Ugandan children [34]. The cellular localisation of these antigenic targets makes them less likely to play a central role in mediating functional immunity, unlike antigens associated with longer-lived antibodies such as *Pf*AMA1, *Pf*MSP1_19_, and EBA175. In settings where most infections are sub-patent and fluctuate above or below the detection limit of PCR, detecting the short-lived antibody responses in individuals or households could imply recent infectivity, particularly in the dry season when clinical cases are rare.

While factors such as the method for determining sero-positivity cut-off values could affect estimates of sero-prevalence or the magnitude of association between endpoints [35], the observations in this study have important implications. In particular, there is a very consistent trend of compound sero-positivity being a risk factor for asymptomatic infection in contrast to being protective against clinical malaria. The complex dynamics between clinical and parasitic immunity are not fully explained through epidemiological data alone as previous studies have observed lower PCR density in areas of higher transmission intensity [36]. Fluctuations in sero-positivity levels may be a strong indicator of a persistent human reservoir of infection, warranting community-targeted interventions if individuals remain infected but are not clinically apparent and continue to transmit. Household serological status could be used for targeting interventions where there are foci of asymptomatically infected individuals.

Immuno-epidemiological studies will be subject to variations in antigen-specific antibody dynamics, as well as the antigenicity of recombinant constructs. Differences in sequence selection and expression systems may result in recombinant proteins that differ slightly from natural epitope conformation or exposure. It is unclear the degree to which these subtle variations affect the strength of antibody detection between recombinant antigens. While this study focuses primarily on total IgG responses, quantification of antibody levels using a combination of isotypes or subclasses may allow for improved detection of sero-incidence. For example, some studies have found that IgG3 antibodies bind more weakly or have a reduced serum half-life relative to IgG1 [37, 38], suggesting that naturally acquired antibody responses can be skewed with respect to isotype and subclass. Studies on switch class variants for both malaria and other infectious pathogens have found that antibody affinity and avidity will differ between antigens of the same pathogen or even between different domains of a single antigen [39, 40]. This can be due to factors such as age and gender, antigen density, strain of the organism, and epitope specificity [38, 41, 42]. Ultimately, current evidence on the associations of antigen-specific IgG subclasses with both exposure and immunity to malaria is varied [43, 44]. Antigens will fall along a continuum of suitability as either biomarkers of acute infection to correlates of protective immunity [28], and these subtleties need to be considered when selecting targets for surveillance. The assays used in this study have allowed us to quantify antibody responses to specific antigens. Therefore, it will also be valuable to investigate the optimal combinations of antigens associated with both short- and long-lived antibodies in surveillance, which could be more routinely used to account for the breadth of antibody response in a population [45–47]. Individual level variations could also be overcome by using multiple variants or chimeric antigen constructs [48].

Changing household sero-prevalence throughout the season may influence the precision of estimates, which could be overcome with more frequent sampling of study participants, such as 3-month intervals depending on antibody decay rates. The findings in this study suggest that household sero-prevalence may be a robust proxy for past malaria exposure. It is simultaneously associated with increased risk of asymptomatic infections, but reduced risk of clinical malaria (potentially due to partial immunity). However, the biological relationship between household serological status and the risk of clinical or parasitic infection could be better investigated and confirmed through controlled observational cohort studies.

Our study findings indicate that a number of new serological markers of malaria exposure could be useful for epidemiological surveillance in highly seasonal malaria transmission areas. There is potential for several of the antigenic targets explored to be used for population-wide screening, all of which have also been optimised for use on a multiplexed bead-based platform in research settings. Further translating this to a lateral flow device may offer opportunities for widespread application if clear use-cases can be established [49]. In light of the strong association between serological status and increased asymptomatic infection, serological platforms may have direct utility as a screening tool in active or reactive case detection strategies, most of which have relied on clinical index cases presenting at health facilities and have shown variability in effectiveness [50]. As malaria transmission declines, surveillance using a diverse panel of antigenic targets can complement existing and emerging diagnostic tools for monitoring changes in transmission. This can include methods such as age-stratified sero-prevalence to estimate sero-conversion rates or measuring age-specific antibody intensity, where estimates of the magnitude of antibody response can often detect more subtle serological changes in the population [51]. Specific monitoring for the presence of individuals or households with higher than age-expected antibody levels, as has been explored in other elimination settings such as Indonesia [52] and South Africa [10], can also be used to identify areas with clustering of high antibody responses as potential reservoirs of infections and guide the spatial targeting of interventions. Serology could also play a role in documenting the absence of malaria transmission, inform baseline risk stratification of study arms in randomised trials, or be used as secondary endpoints in efficacy trials [49]. However, further field testing of these strategies in future studies will be needed, as well as standardised methods for characterising serological responses at the village or cluster level, and determining optimal sampling timeframes or sentinel populations for use in routine surveillance.

## Conclusions

As more countries move towards malaria elimination, including several in sub-Saharan Africa, sensitive surveillance tools will be needed to further understand the dynamics between the parasite, host, and vector at very low levels of transmission. Integrating serological markers of recent exposure into existing field diagnostics and case reporting will become increasingly important in estimating the human reservoir of malaria infection, understanding patterns of transmission, and designing targeted elimination strategies.

## Supplementary information

**Additional file 1.** Sero-prevalence amongst individuals aged 2–10 year, prevalence of sero-positive compounds, and population size and age distribution by compound.

**Additional file 2. **Odds of clinical malaria and asymptomatic infection for sero-positive individuals and individuals residing in sero-positive compounds. Unadjusted and adjusted odds ratios for eight malaria antigenic targets - Etramp5.Ag1, GEXP18, HSP40.Ag1, Rh2.2030, EBA175, *Pf*MSP1_19_, *Pf*AMA1, and *Pf*GLURP.R2.

**Additional file 3.** Map of household geolocation of *Pf*AMA1 sero-positive, clinical malaria and *Pf* infections across four villages during dry and wet transmission seasons. Spatial distribution of infections shown for lower transmission (N’demban) and higher transmission (Besse) villages in the West Coast Region (WCR) and lower transmission (Njaiyal) and higher transmission (Madina Samako) villages in the Upper River Region (URR). Infections at the start of the wet season (June – July 2013) shown on the left and during the wet and transmission season (August – December 2013) on the right.

**Additional file 4.** Gambia serology endpoints study data.

## Data Availability

The datasets used and/or analysed during the current study are provided as an Additional file 4, but personally identifying information on village and compound have been removed to maintain confidentiality. Access to this data can be made available from the corresponding author on reasonable request.

## Abbreviations

aOR: Adjusted odds ratio
CI: Confidence interval
DBS: Dried blood spot
EBA175, RIII-V: Erythrocyte-binding antigen 175 Region III-V
Etramp5.Ag1: Early transcribed membrane protein 5
GEE: Generalised estimating equation
GEXP18: Gametocyte export protein 18
HSP40.Ag1: Heat shock protein 40
IgG: Immunoglobulin
LLIN: Long-lasting insecticide-treated net
MDA: Mass drug administration
MFI: Median fluorescence intensity
PCD: Passive case detection
PCR: Polymerase chain reaction
*Pf, P. falciparum*: *Plasmodium falciparum*
*Pf*AMA1: *P. falciparum* apical membrane antigen 1
*Pf*MSP1_19_: *P. falciparum* merozoite surface antigen 1 19-kDa carboxy-terminal region
RDT: Rapid diagnostic test
Rh2.2030: Reticulocyte binding protein homologue 2
URR: Upper River Region, The Gambia
WCR: West Coast Region, The Gambia
