# Supplementary material for: Antibody responses to a suite of novel serological markers for malaria surveillance demonstrate strong correlation with clinical and parasitological infection across seasons and transmission settings in The Gambia
Source: BMC Med. 2020 Sep 25;18:304. doi: 10.1186/s12916-020-01724-5 (PMC7517687; doi:10.1186/s12916-020-01724-5)

**Table S1. Age 2-10 sero-prevalence and prevalence of sero-positive compounds by region and transmission season.** Sero-positive compounds are defined by any compound with at least one sero-positive individual of any age. 95% confidence intervals (95%CI) are indicated in the parentheses.

| 1. Ages 2-10 years sero-prevalence (95%CI) - short-lived antibody responses | | | | | |
| --- | --- | --- | --- | --- | --- |
|  | **Etramp5.Ag1** | **GEXP18** | **HSP40.Ag1** | **Rh2.2030** | **EBA175** |
| West Coast Region (WCR) | | | | | |
| July 2013 (n=205) | 4.4% (1.6 - 7.2) | 11.7% (7.3 - 16.1) | 5.4% (2.3 - 8.5) | 1.0% (0.0 - 2.3) | 1.5% (0.0 - 3.1) |
| Dec 2013 (n=197) | 13.7% (8.9 - 18.5) | 12.7% (8.0 - 17.3) | 6.1% (2.8 - 9.4) | 1.0% (0.0 - 2.4) | 1.0% (0.0 - 2.4) |
| Upper River Region (URR) | | | | | |
| July 2013 (n=275) | 8.7% (5.4 - 12.1) | 25.1% (20.0 - 30.2) | 8.4% (5.1 - 11.6) | 9.8% (6.3 - 13.3) | 7.3% (4.2 - 10.3) |
| Dec 2013 (n=240) | 33.3% (27.4 - 39.3) | 36.3% (30.2 - 42.3) | 23.8% (18.4 - 29.1) | 25.4% (19.9 - 30.9) | 15.8% (11.2 - 20.5) |
| Apr 2014 (n=324) | 22.8% (18.3 - 27.4) | 34.0% (28.8 - 39.1) | 23.5% (18.8 - 28.1) | 21.9% (17.4 - 26.4) | 13.6% (9.8 - 17.3) |
| Dec 2014 (n=279) | 27.6% (22.4 - 32.8) | 43.4% (37.6 - 49.2) | 27.2% (22.0 - 32.5) | 19.7% (15.0 - 24.4) | 12.5% (8.7 - 16.4) |
| 1. Prevalence of sero-positive compounds - short-lived antibody responses | | | | | |
|  | **Etramp5.Ag1** | **GEXP18** | **HSP40.Ag1** | **Rh2.2030** | **EBA175** |
| West Coast Region (WCR) | | | | | |
| July 2013 (N=87) | 41.4% (25.3 – 57.5) | 55.2% (41.1 – 69.2) | 43.7% (27.9 – 59.4) | 48.3% (33.2 – 63.4) | 63.2% (50.5 – 76.0) |
| Dec 2013 (N=88) | 52.3% (37.8 – 66.7) | 58.0% (44.4 – 71.5) | 46.6% (31.3 – 61.9) | 42.0% (26.1 – 58.0) | 53.4% (39.1 – 67.7) |
| Upper River Region (URR) | | | | | |
| July 2013 (N=54) | 72.2% (58.2 – 86.3) | 90.7% (82.6 – 98.9) | 83.3% (72.4 – 94.2) | 90.7% (82.6 – 98.9) | 92.6% (85.3 – 99.9) |
| Dec 2013 (N=64) | 85.9% (76.8 – 95.1) | 92.2% (85.3 – 99.0) | 79.7% (68.6 – 90.7) | 90.6% (83.1 – 98.1) | 81.2% (70.6 – 91.9) |
| Apr 2014 (N=67) | 85.1% (75.8 – 94.3) | 94.0% (88.2 – 99.9) | 88.1% (79.8 – 96.3) | 95.5% (90.5 – 100) | 91.0% (83.9 – 98.2) |
| Dec 2014 (N=70) | 90.0% (82.6 – 97.4) | 97.1% (93.2 – 100) | 87.1% (78.7 – 95.5) | 92.9% (86.6 – 99.1) | 92.9% (86.6 – 99.1) |
| 1. Ages 2-10 years (95%CI) – long-lived antibody responses | | | | | |
|  | ***Pf*MSP1_19_** | ***Pf*AMA1** | ***Pf*GLURP.R2** |  |  |
| West Coast Region (WCR) | | | | | |
| July 2013 (n=205) | 2.9% (0.6 - 5.2) | 4.4% (1.6 - 7.2) | 4.4% (1.6 - 7.2) |  |  |
| Dec 2013 (n=197) | 8.1% (4.3 - 11.9) | 4.1% (1.3 - 6.8) | 8.1% (4.3 – 11.9) |  |  |
| Upper River Region (URR) | | | | |  |
| July 2013 (n=275) | 5.1% (2.5 - 7.7) | 17.8% (13.3 - 22.3) | 16.4% (12.0 – 20.7) |  |  |
| Dec 2013 (n=240) | 22.1% (16.8 - 27.3) | 33.3% (27.4 - 39.3) | 30.4% (24.6 – 36.2) |  |  |
| Apr 2014 (n=324) | 13.9% (10.1 - 17.7) | 28.4% (23.5 - 33.3) | 28.4% (23.5 – 33.3) |  |  |
| Dec 2014 (n=279) | 15.1% (10.9 - 19.2) | 24.7% (19.7 - 29.8) | 24.4% (19.3 – 29.4) |  |  |
| 1. Prevalence of sero-positive compounds – long-lived antibody responses | | | | | |
|  | ***Pf*MSP1_19_** | ***Pf*AMA1** | ***Pf*GLURP.R2** |  |  |
| West Coast Region (WCR) | | | | | |
| July 2013 (N=87) | 32.2% (14.9 – 49.5) | 77.0% (66.9 – 87.1) | 79.2% (69.8 – 88.9) |  |  |
| Dec 2013 (N=88) | 39.8% (23.6 – 56.0) | 68.2% (56.4 – 80.0) | 80.7% (71.5 – 89.9) |  |  |
| Upper River Region (URR) | | | | | |
| July 2013 (N=54) | 70.4% (55.9 – 84.9) | 96.3% (91.2 - 100) | 96.3% (91.2 - 100) |  |  |
| Dec 2013 (N=64) | 76.6% (62.8 – 87.2) | 95.3% (90.0 - 100) | 96.9% (92.5 - 100) |  |  |
| Apr 2014 (N=67) | 82.1% (72.0 – 92.2) | 98.5% (95.6 - 100) | 98.5% (95.6 - 100) |  |  |
| Dec 2014 (N=70) | 88.6% (80.7 – 96.5) | 98.6% (95.8 - 100) | 100% (100 - 100) |  |  |

**Table S2. Number of compounds in each sero-prevalence range.** Compound is defined as a collection of households centrally located around a main residence. Analysis is limited to households with at least four individuals. Table indicates the total number of unique individuals residing in compounds falling in each sero-prevalence range at any monthly survey during the study.

|  | Compound sero-prevalence 0% | | Compound sero-prevalence 1-50% | | Compound sero-prevalence >50% | |
| --- | --- | --- | --- | --- | --- | --- |
|  | **Individuals** | **Compounds** | **Individuals** | **Compounds** | **Individuals** | **Compounds** |
| Etramp5.Ag1 | 568 | 162 | 1,653 | 143 | 157 | 38 |
| GEXP18 | 394 | 138 | 1,737 | 155 | 310 | 59 |
| HSP40.Ag1 | 567 | 161 | 1,666 | 141 | 139 | 36 |
| Rh2.2030 | 398 | 135 | 1,659 | 140 | 204 | 44 |
| EBA175 | 399 | 130 | 1,680 | 157 | 204 | 47 |
| PfMSP1_19_ | 681 | 170 | 1,568 | 132 | 121 | 34 |
| PfAMA1 | 208 | 98 | 1,681 | 160 | 427 | 80 |
| PfGLURP.R2 | 155 | 82 | 1,708 | 166 | 529 | 95 |

**Figure S1. Compound population size and age range.** Compound is defined as a collection of households centrally located around a main residence. The distribution of individuals per compounds are shown for regions with (A) PCR prevalence less than 15% (West Coast, North Bank, Lower River and Central River Regions), and (B) regions with PCR prevalence greater than 15% (Upper River Regions), as well as the distribution of age ranges and minimum and maximum ages within each compound for all regions (C).


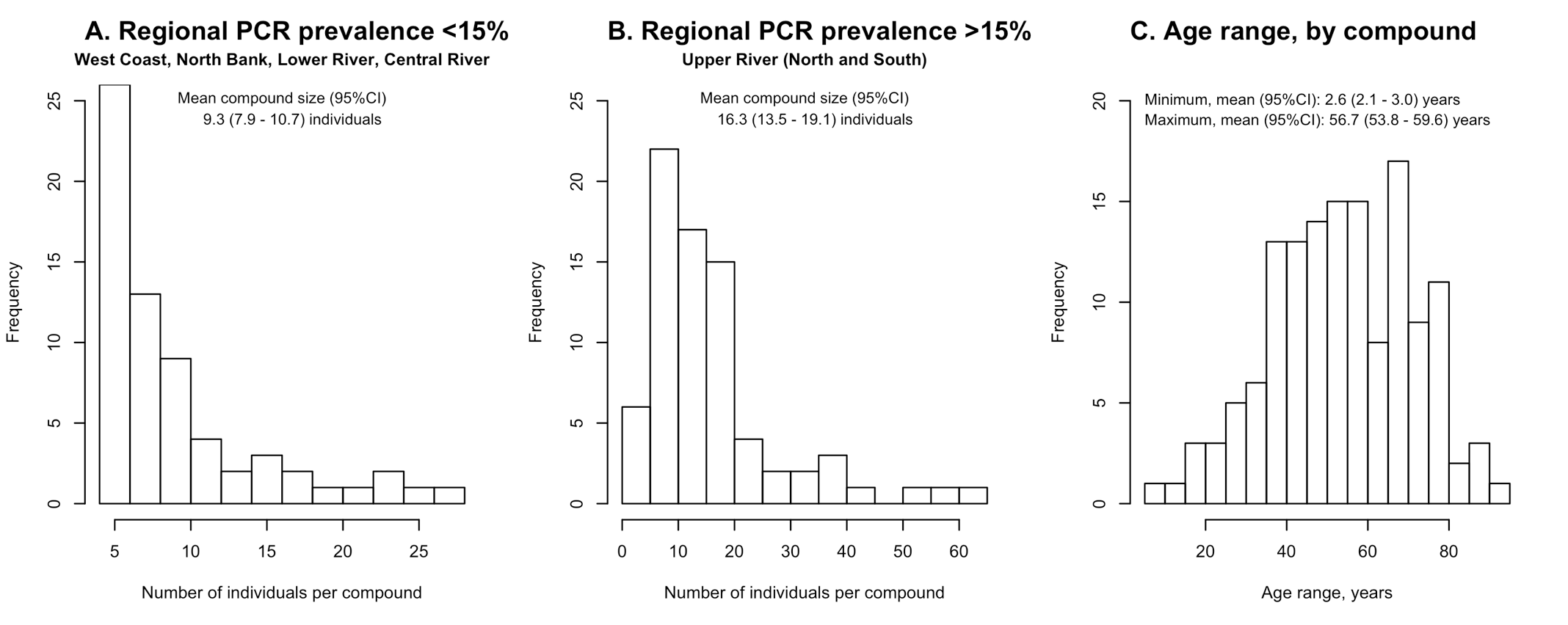

Supplement: Supplementary file 1 — Additional file 1. Sero-prevalence amongst individuals aged 2–10 year, prevalence of sero-positive compounds, and population size and age distribution by compound. [file 12916_2020_1724_MOESM1_ESM.docx]
