# Supplementary material for: Antibody responses to a suite of novel serological markers for malaria surveillance demonstrate strong correlation with clinical and parasitological infection across seasons and transmission settings in The Gambia
Source: BMC Med. 2020 Sep 25;18:304. doi: 10.1186/s12916-020-01724-5 (PMC7517687; doi:10.1186/s12916-020-01724-5)
Supplement: Supplementary file 2 — Additional file 2. Odds of clinical malaria and asymptomatic infection for sero-positive individuals and individuals residing in sero-positive compounds. Unadjusted and adjusted odds ratios for eight malaria antigenic targets - Etramp5.Ag1, GEXP18, HSP40.Ag1, Rh2.2030, EBA175, PfMSP119, PfAMA1, and PfGLURP.R2. [file 12916_2020_1724_MOESM2_ESM.docx]

**Table S3. Odds of clinical malaria amongst Etramp5.Ag1 seropositive individuals and compounds.** Odds of clinical malaria if an individual is concurrently sero-positive to Etramp5.Ag1 is shown unadjusted and adjusted for individual’s age group and LLIN use in the last 24 hours. Odds of clinical malaria if an individual is residing in the same compound as an Etramp5.Ag1 sero-positive individual is shown unadjusted and adjusted for individual’s age group and compound size. Odds of clinical infection based on whether compound sero-prevalence to Etramp5.Ag1 is <50% or >50% is shown unadjusted and adjusted for individual’s age group and LLIN use in the last 24 hours.

| Outcome: Clinical malaria (passive case detection or fever with RDT-positive test) | | |  |  |  |  |
| --- | --- | --- | --- | --- | --- | --- |
|  | **Unadjusted** | | | **Adjusted** | | |
|  | **OR** | **95%CI** | **p-value** | **aOR** | **95%CI** | **p-value** |
| Etramp5.Ag1 positive individual | 5.88 | 3.44 - 10.03 | <0.001 | 4.60 | 2.98 - 7.12 | <0.001 |
| Age group |  |  |  |  |  |  |
| 1-5 years | - | - | - | 1.00 | 1.00 | - |
| 6-15 years | - | - | - | 2.08 | 0.97 - 4.45 | 0.058 |
| > 15 years | - | - | - | 0.75 | 0.35 - 1.61 | 0.460 |
| LLIN use 24 hours | - | - | - | 1.41 | 1.04 - 1.92 | 0.028 |
| Residing in compound with sero-positive individual | 0.19 | 0.03 - 1.06 | 0.059 | 0.17 | 0.03 - 0.89 | 0.036 |
| Age group (individual) | - | - | - | 1.47 | 0.45 - 4.81 | 0.527 |
| Etramp5.Ag1 sero-prevalence compound |  |  |  |  |  |  |
| Sero-prevalence <50% | 0.53 | 0.18 - 1.53 | 0.238 | 0.68 | 0.24 - 1.94 | 0.472 |
| Sero-prevalence >50% | 1.64 | 0.22 - 12.27 | 0.628 | 2.07 | 0.24 - 17.54 | 0.505 |
| Age group (individual) | - | - | - | 1.12 | 0.65 - 1.94 | 0.674 |
| LLIN use 24 hours | - | - | - | 2.22 | 0.61 - 8.08 | 0.228 |

**Table S4. Odds of asymptomatic Pf infection amongst Etramp5.Ag1 seropositive individuals and compounds**. Odds of asymptomatic Pf infection if an individual is concurrently sero-positive to Etramp5.Ag1 is shown as unadjusted and adjusted for individual’s age group and LLIN use in the last 24 hours. Odds of asymptomatic Pf infection if an individual is residing in the same compound as an Etramp5.Ag1 sero-positive individual is shown as unadjusted and adjusted for individual’s age group and compound size. Odds of asymptomatic Pf infection based on whether compound sero-prevalence to Etramp5.Ag1 is <50% or >50% is shown unadjusted and adjusted for individual’s age group and LLIN use in the last 24 hours.

| Outcome: Asymptomatic *Pf* infection (detected by PCR) | | | | | | |
| --- | --- | --- | --- | --- | --- | --- |
|  | **Unadjusted** | | | **Adjusted** | | |
|  | **OR** | **95%CI** | **p-value** | **aOR** | **95%CI** | **p-value** |
| Etramp5.Ag1 positive individual | 3.20 | 2.64 - 3.88 | <0.001 | 3.33 | 2.72 - 4.08 | <0.001 |
| Age group |  |  |  |  |  |  |
| 1-5 years | - | - | - | 1.00 | 1.00 | - |
| 6-15 years | - | - | - | 1.20 | 0.90 - 1.61 | 0.216 |
| > 15 years | - | - | - | 0.82 | 0.58 - 1.15 | 0.250 |
| LLIN use 24 hours | - | - | - | 1.01 | 0.96 - 1.06 | 0.807 |
| Etramp5.Ag1 positive compound | 2.83 | 1.62 - 4.96 | <0.001 | 2.87 | 1.62 - 5.07 | <0.001 |
| Age group (individual) | - | - | - | 1.09 | 0.77 - 1.55 | 0.613 |
| Etramp5.Ag1 sero-prevalence compound |  |  |  |  |  |  |
| Sero-prevalence <50% | 2.35 | 1.42 - 3.90 | 0.001 | 2.52 | 1.49 - 4.28 | 0.001 |
| Sero-prevalence >50% | 7.45 | 4.68 - 11.87 | <0.001 | 8.17 | 5.23 - 12.76 | <0.001 |
| Age group (individual) | - | - | - | 1.10 | 0.76 - 1.59 | 0.614 |
| LLIN use 24 hours | - | - | - | 0.99 | 0.94 - 1.04 | 0.619 |

**Table S5. Odds of clinical malaria amongst Etramp5.Ag1 seropositive individuals.** Odds of clinical malaria if sero-positive to Etramp5.Ag1 is adjusted for individual’s age group and LLIN use in the last 24 hours, allowing for interaction between sero-positivity and age group.

| Outcome: Clinical malaria (passive case detection or fever and RDT+) | | | |
| --- | --- | --- | --- |
|  | **Adjusted** | | |
|  | **aOR** | **95%CI** | **p-value** |
| Etramp5.Ag1 positive individual | 8.35 | 1.72 - 40.55 | 0.008 |
| Age group |  |  |  |
| 1-5 years | 1.00 | 1.00 | - |
| 6-15 years | 3.88 | 1.39 - 10.81 | 0.008 |
| > 15 years | 1.25 | 0.46 - 3.45 | 0.660 |
| LLIN use 24 hours | 1.26 | 1.07 - 1.49 | 0.006 |
| Interaction effects |  |  |  |
| Etramp5.Ag1: Age 6-15 years | 0.91 | 0.12 - 6.88 | 0.928 |
| Etramp5.Ag1: Age >15 years | 0.51 | 0.07 - 3.76 | 0.512 |

**Table S6. Odds of asymptomatic Pf infection amongst Etramp5.Ag1 seropositive individuals.** Odds of asymptomatic Pf infection if sero-positive to Etramp5.Ag1 is adjusted for individual’s age group and LLIN use in the last 24 hours, allowing for interaction between sero-positivity and age group.

| Outcome: Asymptomatic *Pf* infection (detected by PCR) | | | |
| --- | --- | --- | --- |
|  | **Adjusted** | | |
|  | **aOR** | **95%CI** | **p-value** |
| Etramp5.Ag1 positive individual | 4.81 | 3.16 - 7.34 | <0.001 |
| Age group |  |  |  |
| 1-5 years | 1.00 | 1.00 | - |
| 6-15 years | 1.29 | 0.92 - 1.80 | 0.139 |
| > 15 years | 1.09 | 0.76 - 1.57 | 0.636 |
| LLIN use 24 hours | 1.01 | 0.96 - 1.06 | 0.840 |
| Interaction effects |  |  |  |
| Etramp5.Ag1: Age 6-15 years | 0.78 | 0.47 - 1.31 | 0.352 |
| Etramp5.Ag1: Age >15 years | 0.50 | 0.30 - 0.82 | 0.006 |

**Table S7. Odds of clinical malaria amongst GEXP18 seropositive individuals and compounds.** Odds of clinical malaria if an individual is concurrently sero-positive to GEXP18 is shown as unadjusted and adjusted for individual’s age group and LLIN use in the last 24 hours. Odds of clinical malaria if an individual is residing in the same compound as a GEXP18 sero-positive individual is shown as unadjusted and adjusted for individual’s age group and compound size. Odds of clinical malaria based on whether compound sero-prevalence to GEXP18 is <50% or >50% is shown unadjusted and adjusted for individual’s age group and LLIN use in the last 24 hours.

| Outcome: Clinical malaria (passive case detection or fever and RDT+) | | |  |  |  |  |
| --- | --- | --- | --- | --- | --- | --- |
|  | **Unadjusted** | | | **Adjusted** | | |
|  | **OR** | **95%CI** | **p-value** | **OR** | **95%CI** | **p-value** |
| GEXP18 positive individual | 2.57 | 1.44 - 4.61 | 0.002 | 1.48 | 0.90 - 2.41 | 0.122 |
| Age group |  |  |  |  |  |  |
| 1-5 years | - | - | - | 1.00 | 1.00 | - |
| 6-15 years | - | - | - | 2.43 | 1.13 - 5.23 | 0.023 |
| > 15 years | - | - | - | 0.99 | 0.46 - 2.13 | 0.980 |
| LLIN use 24 hours | - | - | - | 1.35 | 1.05 - 1.73 | 0.018 |
| Residing in compound with sero-positive individual | 0.13 | 0.02 - 0.90 | 0.039 | 0.11 | 0.02 - 0.73 | 0.022 |
| Age group (individual) | - | - | - | 1.50 | 0.44 - 5.04 | 0.517 |
| GEXP18 sero-prevalence compound |  |  |  |  |  |  |
| Sero-prevalence <25% | 0.77 | 0.13 - 4.65 | 0.779 | 0.78 | 0.15 - 3.97 | 0.766 |
| Sero-prevalence >50% | 1.26 | 0.13 - 12.32 | 0.841 | 2.21 | 0.26 - 18.80 | 0.467 |
| Age group (individual) | - | - | - | 1.09 | 0.66 - 1.94 | 0.665 |
| LLIN use 24 hours | - | - | - | 1.82 | 0.62 - 10.06 | 0.200 |

**Table S8. Odds of asymptomatic Pf infection amongst GEXP18 seropositive individuals and compounds.** Odds of asymptomatic Pf infection if an individual is concurrently sero-positive to GEXP18 is shown as unadjusted and adjusted for individual’s age group and LLIN use in the last 24 hours. Odds of asymptomatic Pf infection if an individual is residing in the same compound as an GEXP18 sero-positive individual is shown as unadjusted and adjusted for individual’s age group and compound size. Odds of asymptomatic infection based on whether compound sero-prevalence to GEXP18 is <50% or >50% is shown unadjusted and adjusted for individual’s age group and LLIN use in the last 24 hours.

| Outcome: Asymptomatic *Pf* infection (detected by PCR) | | | | | | |
| --- | --- | --- | --- | --- | --- | --- |
|  | **Unadjusted** | | | **Adjusted** | | |
|  | **OR** | **95%CI** | **p-value** | **aOR** | **95%CI** | **p-value** |
| GEXP18 positive individual | 2.94 | 2.38 - 3.64 | <0.001 | 3.12 | 2.50 - 3.90 | <0.001 |
| Age group |  |  |  |  |  |  |
| 1-5 years | - | - | - | 1.00 | 1.00 | - |
| 6-15 years | - | - | - | 1.39 | 1.03 - 1.86 | 0.030 |
| > 15 years | - | - | - | 0.97 | 0.68 - 1.36 | 0.841 |
| LLIN use 24 hours | - | - | - | 1.02 | 0.97 - 1.07 | 0.434 |
| GEXP18 positive compound | 2.65 | 1.58 - 4.47 | <0.001 | 2.61 | 1.54 - 4.42 | <0.001 |
| Age group (individual) | - | - | - | 1.10 | 0.77 - 1.56 | 0.598 |
| GEXP18 sero-prevalence compound |  |  |  |  |  |  |
| Sero-prevalence <50% | 2.48 | 1.43 - 4.32 | 0.001 | 2.86 | 1.44 - 5.69 | 0.003 |
| Sero-prevalence >50% | 7.41 | 3.24 - 16.96 | <0.001 | 8.85 | 3.54 - 22.08 | <0.001 |
| Age group (individual) | - | - | - | 1.11 | 0.78 - 1.59 | 0.551 |
| LLIN use 24 hours | - | - | - | 0.99 | 0.94 - 1.05 | 0.770 |

**Table S9. Odds of clinical malaria amongst GEXP18 seropositive individuals.** Odds of clinical malaria if sero-positive to GEXP18 is adjusted for individual’s age group and LLIN use in the last 24 hours, allowing for interaction between sero-positivity and age group.

| Outcome: Clinical malaria (passive case detection or fever and RDT+) | | | |
| --- | --- | --- | --- |
|  | **Adjusted** | | |
|  | **aOR** | **95%CI** | **p-value** |
| GEXP18 positive individual | 1.57 | 0.55 - 4.45 | 0.396 |
| Age group |  |  |  |
| 1-5 years | 1.00 | 1.00 | - |
| 6-15 years | 4.54 | 1.77 - 11.67 | 0.002 |
| > 15 years | 0.85 | 0.30 - 2.39 | 0.762 |
| LLIN use 24 hours | 1.34 | 1.13 - 1.59 | 0.001 |
| Interaction effects |  |  |  |
| GEXP18: Age 6-15 years | 1.30 | 0.26 - 6.46 | 0.745 |
| GEXP18: Age >15 years | 4.09 | 0.94 - 17.81 | 0.061 |

**Table S10. Odds of asymptomatic Pf infection amongst GEXP18 seropositive individuals.** Odds of asymptomatic Pf infection if sero-positive to Etramp5.Ag1 is adjusted for individual’s age group and LLIN use in the last 24 hours, allowing for interaction between sero-positivity and age group.

| Outcome: Asymptomatic *Pf* infection (detected by PCR) | | | |
| --- | --- | --- | --- |
|  | **Adjusted** | | |
|  | **aOR** | **95%CI** | **p-value** |
| GEXP18 positive individual | 3.06 | 1.90 - 4.92 | <0.001 |
| Age group |  |  |  |
| 1-5 years | 1.00 | 1.00 | - |
| 6-15 years | 1.20 | 0.82 - 1.77 | 0.344 |
| > 15 years | 1.13 | 0.76 - 1.68 | 0.539 |
| LLIN use 24 hours | 1.02 | 0.97 - 1.07 | 0.441 |
| Interaction effects |  |  |  |
| GEXP18: Age 6-15 years | 1.32 | 0.76 - 2.30 | 0.327 |
| GEXP18: Age >15 years | 0.75 | 0.43 - 1.32 | 0.323 |

**Table S11. Odds of clinical malaria amongst HSP40.Ag1 seropositive individuals and compounds.** Odds of clinical malaria if an individual is concurrently sero-positive to HSP40.Ag1 is shown as unadjusted and adjusted for individual’s age group and LLIN use in the last 24 hours. Odds of clinical malaria if an individual is residing in the same compound as a HSP40.Ag1 sero-positive individual is shown as unadjusted and adjusted for individual’s age group and compound size. Odds of clinical malaria based on whether compound sero-prevalence to HSP40.Ag1 is <50% or >50% is shown unadjusted and adjusted for individual’s age group and LLIN use in the last 24 hours.

| Outcome: Clinical malaria (passive case detection or fever and RDT+) | | |  |  |  |  |
| --- | --- | --- | --- | --- | --- | --- |
|  | **Unadjusted** | | | **Adjusted** | | |
|  | **OR** | **95%CI** | **p-value** | **OR** | **95%CI** | **p-value** |
| HSP40.Ag1 positive individual | 1.67 | 0.88 - 3.18 | 0.119 | 0.99 | 0.59 - 1.65 | 0.956 |
| Age group |  |  |  |  |  |  |
| 1-5 years | - | - | - | 1.00 | 1.00 | - |
| 6-15 years | - | - | - | 2.40 | 1.11 - 5.20 | 0.026 |
| > 15 years | - | - | - | 1.01 | 0.46 - 2.24 | 0.973 |
| LLIN use 24 hours | - | - | - | 1.32 | 1.04 - 1.68 | 0.020 |
| Residing in compound with sero-positive individual | 0.14 | 0.02 - 0.80 | 0.027 | 0.13 | 0.02 - 0.70 | 0.018 |
| Age group (individual) | - | - | - | 1.46 | 0.47 - 4.54 | 0.511 |
| HSP40.Ag1 sero-prevalence compound |  |  |  |  |  |  |
| Sero-prevalence <50% | 0.31 | 0.10 - 1.00 | 0.051 | 0.42 | 0.13 - 1.33 | 0.139 |
| Sero-prevalence >50% | 1.70 | 0.16 - 18.28 | 0.660 | 2.37 | 0.22 - 25.63 | 0.477 |
| Age group (individual) | - | - | - | 1.14 | 0.67 - 1.92 | 0.637 |
| LLIN use 24 hours | - | - | - | 2.09 | 0.57 - 7.62 | 0.266 |

**Table S12. Odds of asymptomatic Pf infection amongst HSP40.Ag1 seropositive individuals and compounds.** Odds of asymptomatic Pf infection if an individual is concurrently sero-positive to HSP40.Ag1 is shown as unadjusted and adjusted for individual’s age group and LLIN use in the last 24 hours. Odds of asymptomatic Pf infection if an individual is residing in the same compound as an HSP40.Ag1 sero-positive individual is shown as unadjusted and adjusted for individual’s age group and compound size. Odds of asymptomatic infection based on whether compound sero-prevalence to HSP40.Ag1 is <50% or >50% is shown unadjusted and adjusted for individual’s age group and LLIN use in the last 24 hours.

| Outcome: Asymptomatic *Pf* infection (detected by PCR) | | | | | |  |
| --- | --- | --- | --- | --- | --- | --- |
|  | **Unadjusted** | | | **Adjusted** | | |
|  | **OR** | **95%CI** | **p-value** | **aOR** | **95%CI** | **p-value** |
| HSP40.Ag1 positive individual | 2.53 | 2.06 - 3.10 | <0.001 | 2.64 | 2.09 - 3.33 | <0.001 |
| Age group |  |  |  |  |  |  |
| 1-5 years | - | - | - | 1.00 | 1.00 | - |
| 6-15 years | - | - | - | 1.22 | 0.91 - 1.63 | 0.175 |
| > 15 years | - | - | - | 0.83 | 0.58 - 1.17 | 0.289 |
| LLIN use 24 hours | - | - | - | 1.01 | 0.96 - 1.06 | 0.815 |
| HSP40.Ag1 positive compound | 1.32 | 0.77 - 2.28 | 0.316 | 1.38 | 0.79 - 2.41 | 0.257 |
| Age group (individual) | - | - | - | 1.10 | 0.77 - 1.56 | 0.600 |
| HSP40.Ag1 sero-prevalence compound |  |  |  |  |  |  |
| Sero-prevalence <50% | 1.45 | 0.87 - 2.43 | 0.153 | 1.45 | 0.85 - 2.50 | 0.176 |
| Sero-prevalence >50% | 3.78 | 2.05 – 6.96 | <0.001 | 3.98 | 2.16 - 7.34 | <0.001 |
| Age group (individual) | - | - | - | 1.10 | 0.76 - 1.58 | 0.614 |
| LLIN use 24 hours | - | - | - | 0.98 | 0.94 - 1.04 | 0.559 |

**Table S13. Odds of clinical malaria amongst HSP40.Ag1 seropositive individuals.** Odds of clinical malaria if sero-positive to HSP40.Ag1 is adjusted for individual’s age group and LLIN use in the last 24 hours, allowing for interaction between sero-positivity and age group.

| Outcome: Clinical malaria (passive case detection or fever and RDT+) | | | |
| --- | --- | --- | --- |
|  | **Adjusted** | | |
|  | **aOR** | **95%CI** | **p-value** |
| HSP40.Ag1 positive individual | 4.95 | 1.06 - 23.17 | 0.042 |
| Age group |  |  |  |
| 1-5 years | 1.00 | 1.00 | - |
| 6-15 years | 6.49 | 2.63 - 16.01 | <0.001 |
| > 15 years | 1.45 | 0.56 - 3.74 | 0.446 |
| LLIN use 24 hours | 1.29 | 1.10 - 1.51 | 0.002 |
| Interaction effects |  |  |  |
| HSP40.Ag1: Age 6-15 years | 0.17 | 0.03 - 0.99 | 0.049 |
| HSP40.Ag1: Age >15 years | 0.48 | 0.08 - 2.92 | 0.423 |

**Table S14. Odds of asymptomatic Pf infection amongst HSP40.Ag1 seropositive individuals.** Odds of asymptomatic infection if sero-positive to HSP40.Ag1 is adjusted for individual’s age group and LLIN use in the last 24 hours, allowing for interaction between sero-positivity and age group.

| Outcome: Asymptomatic *Pf* infection (detected by PCR) | | |  |
| --- | --- | --- | --- |
|  | **Adjusted** | | |
|  | **aOR** | **95%CI** | **p-value** |
| HSP40.Ag1 positive individual | 4.78 | 2.91 - 7.86 | <0.001 |
| Age group |  |  |  |
| 1-5 years | 1.00 | 1.00 | - |
| 6-15 years | 1.35 | 0.98 - 1.86 | 0.064 |
| > 15 years | 1.16 | 0.83 - 1.61 | 0.385 |
| LLIN use 24 hours | 1.00 | 0.96 - 1.05 | 0.881 |
| Interaction effects |  |  |  |
| HSP40.Ag1: Age 6-15 years | 0.64 | 0.35 - 1.15 | 0.134 |
| HSP40.Ag1: Age >15 years | 0.36 | 0.22 - 0.62 | <0.001 |

**Table S15. Odds of clinical malaria amongst Rh2.2030 seropositive individuals and compounds.** Odds of clinical malaria if an individual is concurrently sero-positive to Rh2.2030 is shown as unadjusted and adjusted for individual’s age group and LLIN use in the last 24 hours. Odds of clinical malaria if an individual is residing in the same compound as a Rh2.2030 sero-positive individual is shown as unadjusted and adjusted for individual’s age group and compound size. Odds of clinical malaria based on whether compound sero-prevalence to Rh2.2030 is <50% or >50% is shown unadjusted and adjusted for individual’s age group and LLIN use in the last 24 hours.

| Outcome: Clinical malaria (passive case detection or fever and RDT+) | | |  |  |  |  |
| --- | --- | --- | --- | --- | --- | --- |
|  | **Unadjusted** | | | **Adjusted** | | |
|  | **OR** | **95%CI** | **p-value** | **OR** | **95%CI** | **p-value** |
| Rh2.2030 positive individual | 2.20 | 1.10 - 4.38 | 0.025 | 1.28 | 0.77 - 2.02 | 0.338 |
| Age group |  |  |  |  |  |  |
| 1-5 years | - | - | - | 1.00 | 1.00 | - |
| 6-15 years | - | - | - | 2.32 | 1.08 – 4.99 | 0.031 |
| > 15 years | - | - | - | 0.92 | 0.43 - 1.93 | 0.817 |
| LLIN use 24 hours | - | - | - | 1.34 | 1.04 - 1.72 | 0.023 |
| Residing in compound with sero-positive individual | 0.07 | 0.01 - 0.48 | 0.006 | 0.06 | 0.01 - 0.35 | 0.002 |
| Age group (individual) | - | - | - | 1.65 | 0.60 - 4.57 | 0.332 |
| Rh2.2030 sero-prevalence compound |  |  |  |  |  |  |
| Sero-prevalence <50% | 0.24 | 0.08 - 0.69 | 0.008 | 0.29 | 0.10 - 0.80 | 0.017 |
| Sero-prevalence >50% | 0.67 | 0.08 - 5.99 | 0.721 | 0.77 | 0.08 - 7.64 | 0.823 |
| Age group (individual) | - | - | - | 1.17 | 0.69 - 1.99 | 0.556 |
| LLIN use 24 hours | - | - | - | 2.14 | 0.59 –-7.73 | 0.244 |

**Table S16. Odds of asymptomatic Pf infection amongst Rh2.2030 seropositive individuals and compounds.** Odds of asymptomatic Pf infection if an individual is concurrently sero-positive to Rh2.2030 is shown as unadjusted and adjusted for individual’s age group and LLIN use in the last 24 hours. Odds of asymptomatic Pf infection if an individual is residing in the same compound as an Rh2.2030 sero-positive individual is shown as unadjusted and adjusted for individual’s age group and compound size. Odds of asymptomatic infection based on whether compound sero-prevalence to Rh2.2030 is <50% or >50% is shown unadjusted and adjusted for individual’s age group and LLIN use in the last 24 hours.

| Outcome: Asymptomatic *Pf* infection (detected by PCR) | | |  |  |  |  |
| --- | --- | --- | --- | --- | --- | --- |
|  | **Unadjusted** | | | **Adjusted** | | |
|  | **OR** | **95%CI** | **p-value** | **aOR** | **95%CI** | **p-value** |
| Rh2.2030 positive individual | 2.45 | 1.98 - 3.03 | <0.001 | 3.06 | 2.40 - 3.89 | <0.001 |
| Age group |  |  |  |  |  |  |
| 1-5 years | - | - | - | 1.00 | 1.00 | - |
| 6-15 years | - | - | - | 1.09 | 0.81 - 1.47 | 0.570 |
| > 15 years | - | - | - | 0.62 | 0.43 - 0.89 | 0.009 |
| LLIN use 24 hours | - | - | - | 1.01 | 0.96 - 1.06 | 0.687 |
| Rh2.2030 positive compound | 1.69 | 0.96 - 2.99 | 0.070 | 1.92 | 1.10 - 3.36 | 0.022 |
| Age group (individual) | - | - | - | 1.10 | 0.77 - 1.56 | 0.599 |
| Rh2.2030 sero-prevalence compound |  |  |  |  |  |  |
| Sero-prevalence <50% | 1.78 | 1.01 - 3.13 | 0.047 | 2.00 | 1.13 - 3.53 | 0.017 |
| Sero-prevalence >50% | 4.55 | 2.60 – 7.98 | <0.001 | 5.15 | 2.96 - 8.98 | <0.001 |
| Age group (individual) | - | - | - | 1.10 | 0.76 - 1.58 | 0.624 |
| LLIN use 24 hours | - | - | - | 0.98 | 0.93 - 1.03 | 0.498 |

**Table S17. Odds of clinical malaria amongst Rh2.2030 seropositive individuals.** Odds of clinical malaria if sero-positive to Rh2.2030 is adjusted for individual’s age group and LLIN use in the last 24 hours, allowing for interaction between sero-positivity and age group.

| Outcome: Clinical malaria (passive case detection or fever and RDT+) | | | |
| --- | --- | --- | --- |
|  | **Adjusted** | | |
|  | **aOR** | **95%CI** | **p-value** |
| Rh2.2030 positive individual | 11.90 | 1.69 - 83.80 | 0.013 |
| Age group |  |  |  |
| 1-5 years | 1.00 | 1.00 | - |
| 6-15 years | 4.73 | 1.91 - 11.71 | 0.001 |
| > 15 years | 0.76 | 0.28 - 2.02 | 0.578 |
| LLIN use 24 hours | 1.33 | 1.13 - 1.56 | 0.001 |
| Interaction effects |  |  |  |
| Rh2.2030: Age 6-15 years | 0.11 | 0.01 - 0.97 | 0.047 |
| Rh2.2030: Age >15 years | 0.36 | 0.04 - 3.45 | 0.377 |

**Table S18. Odds of asymptomatic Pf infection amongst Rh2.2030 seropositive individuals.** Odds of asymptomatic Pf infection if sero-positive to Rh2.2030 is adjusted for individual’s age group and LLIN use in the last 24 hours, allowing for interaction between sero-positivity and age group.

| Outcome: Asymptomatic *Pf* infection (detected by PCR) | | | |
| --- | --- | --- | --- |
|  | **Adjusted** | | |
|  | **aOR** | **95%CI** | **p-value** |
| Rh2.2030 positive individual | 4.98 | 2.42 - 10.23 | <0.001 |
| Age group |  |  |  |
| 1-5 years | 1.00 | 1.00 | - |
| 6-15 years | 1.08 | 0.76 - 1.52 | 0.677 |
| > 15 years | 0.89 | 0.61 - 1.29 | 0.529 |
| LLIN use 24 hours | 1.01 | 0.96 - 1.06 | 0.643 |
| Interaction effects |  |  |  |
| Rh2.2030: Age 6-15 years | 0.77 | 0.35 - 1.71 | 0.527 |
| Rh2.2030: Age >15 years | 0.39 | 0.18 - 0.84 | 0.016 |

**Table S19. Odds of clinical malaria amongst EBA175 seropositive individuals and compounds.** Odds of clinical malaria if an individual is concurrently sero-positive to EBA175 is shown as unadjusted and adjusted for individual’s age group and LLIN use in the last 24 hours. Odds of clinical malaria if an individual is residing in the same compound as an EBA175 sero-positive individual is shown as unadjusted and adjusted for individual’s age group and compound size. Odds of clinical malaria based on whether compound sero-prevalence to EBA175 is <50% or >50% is shown unadjusted and adjusted for individual’s age group and LLIN use in the last 24 hours.

| Outcome: Clinical malaria (passive case detection or fever and RDT+) | | |  |  |  |  |
| --- | --- | --- | --- | --- | --- | --- |
|  | **Unadjusted** | | | **Adjusted** | | |
|  | **OR** | **95%CI** | **p-value** | **OR** | **95%CI** | **p-value** |
| EBA175 positive individual | 1.37 | 0.67 - 2.81 | 0.390 | 1.54 | 0.86 - 2.75 | 0.147 |
| Age group |  |  |  |  |  |  |
| 1-5 years | - | - | - | 1.00 | 1.00 | - |
| 6-15 years | - | - | - | 2.31 | 1.09 - 4.91 | 0.030 |
| > 15 years | - | - | - | 0.82 | 0.39 - 1.72 | 0.601 |
| LLIN use 24 hours | - | - | - | 1.34 | 1.05 - 1.72 | 0.021 |
| Residing in compound with sero-positive individual | 0.07 | 0.01 - 0.61 | 0.016 | 0.07 | 0.01 - 0.57 | 0.013 |
| Age group (individual) | - | - | - | 1.31 | 0.46 - 3.72 | 0.606 |
| EBA175 sero-prevalence compound |  |  |  |  |  |  |
| Sero-prevalence <50% | 0.10 | 0.02 - 0.52 | 0.006 | 0.12 | 0.02 - 0.65 | 0.013 |
| Sero-prevalence >50% | 0.41 | 0.03 - 5.21 | 0.493 | 0.52 | 0.04 - 6.52 | 0.609 |
| Age group (individual) | - | - | - | 1.11 | 0.65 - 1.90 | 0.699 |
| LLIN use 24 hours | - | - | - | 1.92 | 0.57 – 6.50 | 0.296 |

**Table S20. Odds of asymptomatic Pf infection amongst EBA175 seropositive individuals and compounds.** Odds of asymptomatic Pf infection if an individual is concurrently sero-positive to EBA175 is shown as unadjusted and adjusted for individual’s age group and LLIN use in the last 24 hours. Odds of asymptomatic Pf infection if an individual is residing in the same compound as an EBA175 sero-positive individual is shown as unadjusted and adjusted for individual’s age group and compound size. Odds of asymptomatic infection based on whether compound sero-prevalence to EBA175 is <50% or >50% is shown unadjusted and adjusted for individual’s age group and LLIN use in the last 24 hours.

| Outcome: Asymptomatic *Pf* infection (detected by PCR) | | | | | |  |
| --- | --- | --- | --- | --- | --- | --- |
|  | **Unadjusted** | | | **Adjusted** | | |
|  | **OR** | **95%CI** | **p-value** | **aOR** | **95%CI** | **p-value** |
| EBA175 positive individual | 2.09 | 1.74 - 2.51 | <0.001 | 2.86 | 2.21 - 3.72 | <0.001 |
| Age group |  |  |  |  |  |  |
| 1-5 years | - | - | - | 1.00 | 1.00 | - |
| 6-15 years | - | - | - | 1.19 | 0.89 - 1.58 | 0.245 |
| > 15 years | - | - | - | 0.59 | 0.40 - 0.88 | 0.009 |
| LLIN use 24 hours | - | - | - | 1.01 | 0.96 - 1.06 | 0.773 |
| EBA175 positive compound | 1.00 | 0.50 - 2.01 | 0.992 | 1.02 | 0.50 - 2.09 | 0.954 |
| Age group (individual) | - | - | - | 1.10 | 0.77 - 1.56 | 0.593 |
| EBA175 sero-prevalence compound |  |  |  |  |  |  |
| Sero-prevalence <50% | 1.11 | 0.62 - 1.98 | 0.717 | 1.06 | 0.58 - 1.95 | 0.843 |
| Sero-prevalence >50% | 2.38 | 1.15 - 4.95 | 0.020 | 2.48 | 1.16 - 5.30 | 0.020 |
| Age group (individual) | - | - | - | 1.10 | 0.76 - 1.58 | 0.613 |
| LLIN use 24 hours | - | - | - | 0.98 | 0.93 - 1.03 | 0.493 |

**Table S21. Odds of clinical malaria amongst EBA175 seropositive individuals.** Odds of clinical malaria if sero-positive to EBA175 is adjusted for individual’s age group and LLIN use in the last 24 hours, allowing for interaction between sero-positivity and age group.

| Outcome: Clinical malaria (passive case detection or fever and RDT+) | | | |
| --- | --- | --- | --- |
|  | **Adjusted** | | |
|  | **aOR** | **95%CI** | **p-value** |
| EBA175 positive individual | 0.04 | 0.00 - 0.49 | 0.012 |
| Age group |  |  |  |
| 1-5 years | 1.00 | 1.00 | - |
| 6-15 years | 4.21 | 1.72 - 10.31 | 0.002 |
| > 15 years | 0.81 | 0.28 - 2.34 | 0.691 |
| LLIN use 24 hours | 1.34 | 1.12 - 1.60 | 0.001 |
| Interaction effects |  |  |  |
| EBA175: Age 6-15 years | 78.05 | 3.84 - 1,586 | 0.005 |
| EBA175: Age >15 years | 68.41 | 3.65 - 1,280 | 0.005 |

**Table S22. Odds of asymptomatic Pf infection amongst EBA175 seropositive individuals.** Odds of asymptomatic infection if sero-positive to EBA175 is adjusted for individual’s age group and LLIN use in the last 24 hours, allowing for interaction between sero-positivity and age group.

| Outcome: Asymptomatic *Pf* infection (detected by PCR) | | | |
| --- | --- | --- | --- |
|  | **Adjusted** | | |
|  | **aOR** | **95%CI** | **p-value** |
| EBA175 positive individual | 3.50 | 2.05 - 5.99 | <0.001 |
| Age group |  |  |  |
| 1-5 years | 1.00 | 1.00 | - |
| 6-15 years | 1.09 | 0.80 - 1.49 | 0.593 |
| > 15 years | 0.84 | 0.56 - 1.26 | 0.397 |
| LLIN use 24 hours | 1.01 | 0.96 - 1.06 | 0.761 |
| Interaction effects |  |  |  |
| EBA175: Age 6-15 years | 1.21 | 0.68 - 2.14 | 0.518 |
| EBA175: Age >15 years | 0.50 | 0.28 - 0.90 | 0.022 |

**Table S23. Odds of clinical malaria amongst PfMSP1_19_ seropositive individuals and compounds.** Odds of clinical malaria if an individual is concurrently sero-positive to PfMSP1_19_ is shown as unadjusted and adjusted for individual’s age group and LLIN use in the last 24 hours. Odds of clinical malaria if an individual is residing in the same compound as a PfMSP1_19_ sero-positive individual is shown as unadjusted and adjusted for individual’s age group and compound size. Odds of clinical malaria based on whether compound sero-prevalence to PfMSP1_19_ is <50% or >50% is shown unadjusted and adjusted for individual’s age group and LLIN use in the last 24 hours.

| Outcome: Clinical malaria (passive case detection or fever and RDT+) | | |  |  |  |  |
| --- | --- | --- | --- | --- | --- | --- |
|  | **Unadjusted** | | | **Adjusted** | | |
|  | **OR** | **95%CI** | **p-value** | **OR** | **95%CI** | **p-value** |
| *Pf*MSP1_19_ positive individual | 3.83 | 1.95 - 7.51 | <0.001 | 4.09 | 2.60 - 6.44 | <0.001 |
| Age group |  |  |  |  |  |  |
| 1-5 years | - | - | - | 1.00 | 1.00 | - |
| 6-15 years | - | - | - | 2.12 | 1.01 - 4.46 | 0.047 |
| > 15 years | - | - | - | 0.68 | 0.31 - 1.49 | 0.331 |
| LLIN use 24 hours | - | - | - | 1.39 | 1.05 - 1.84 | 0.022 |
| Residing in compound with sero-positive individual | 0.15 | 0.03 - 0.87 | 0.034 | 0.12 | 0.03 - 0.50 | 0.004 |
| Age group (individual) | - | - | - | 1.75 | 0.63 - 4.87 | 0.282 |
| *Pf*MSP1_19_ sero-prevalence compound |  |  |  |  |  |  |
| Sero-prevalence <50% | 0.29 | 0.10 - 0.84 | 0.023 | 0.36 | 0.12 - 1.08 | 0.068 |
| Sero-prevalence >50% | 2.17 | 0.22 - 21.83 | 0.509 | 3.01 | 0.27 - 34.90 | 0.377 |
| Age group (individual) | - | - | - | 1.17 | 0.70 - 1.97 | 0.543 |
| LLIN use 24 hours | - | - | - | 2.10 | 0.58 - 7.56 | 0.257 |

**Table S24. Odds of asymptomatic Pf infection amongst PfMSP1_19_ seropositive individuals and compounds.** Odds of asymptomatic Pf infection if an individual is concurrently sero-positive to PfMSP1_19_ is shown as unadjusted and adjusted for individual’s age group and LLIN use in the last 24 hours. Odds of asymptomatic Pf infection if an individual is residing in the same compound as an PfMSP1_19_ sero-positive individual is shown as unadjusted and adjusted for individual’s age group and compound size. Odds of asymptomatic infection based on whether compound sero-prevalence to PfMSP1_19_ is <50% or >50% is shown unadjusted and adjusted for individual’s age group and LLIN use in the last 24 hours.

| Outcome: Asymptomatic *Pf* infection (detected by PCR) | | | | |  |  |
| --- | --- | --- | --- | --- | --- | --- |
|  | **Unadjusted** | | | **Adjusted** | | |
|  | **OR** | **95%CI** | **p-value** | **aOR** | **95%CI** | **p-value** |
| *Pf*MSP1_19_ positive individual | 2.29 | 1.82 - 2.88 | <0.001 | 2.49 | 1.90 - 3.27 | <0.001 |
| Age group |  |  |  |  |  |  |
| 1-5 years | - | - | - | 1.00 | 1.00 | - |
| 6-15 years | - | - | - | 1.24 | 0.93 - 1.66 | 0.138 |
| > 15 years | - | - | - | 0.81 | 0.56 - 1.17 | 0.263 |
| LLIN use 24 hours | - | - | - | 1.00 | 0.96 - 1.05 | 0.932 |
| *Pf*MSP1_19_ positive compound | 1.87 | 1.15 - 3.06 | 0.012 | 1.95 | 1.19 - 3.20 | 0.008 |
| Age group (individual) | - | - | - | 1.10 | 0.77 - 1.56 | 0.608 |
| *Pf*MSP1_19_ sero-prevalence compound |  |  |  |  |  |  |
| Sero-prevalence <50% | 1.97 | 1.24 - 3.12 | 0.004 | 2.13 | 1.30 - 3.50 | 0.003 |
| Sero-prevalence >50% | 7.53 | 4.42 - 12.83 | <0.001 | 8.75 | 4.96 - 15.43 | <0.001 |
| Age group (individual) | - | - | - | 1.10 | 0.76 - 1.58 | 0.619 |
| LLIN use 24 hours | - | - | - | 0.99 | 0.94 - 1.04 | 0.637 |

**Table S25. Odds of clinical malaria amongst PfMSP1_19_ seropositive individuals.** Odds of clinical malaria if sero-positive to PfMSP1_19_ is adjusted for individual’s age group and LLIN use in the last 24 hours, allowing for interaction between sero-positivity and age group.

| Outcome: Clinical malaria (passive case detection or fever and RDT+) | | | |
| --- | --- | --- | --- |
|  | **Adjusted** | | |
|  | **aOR** | **95%CI** | **p-value** |
| *Pf*MSP1_19_ positive individual | 3.77 | 0.72 - 19.75 | 0.117 |
| Age group |  |  |  |
| 1-5 years | 1.00 | 1.00 | - |
| 6-15 years | 3.57 | 1.42 - 8.97 | 0.007 |
| > 15 years | 1.28 | 0.52 - 3.13 | 0.594 |
| LLIN use 24 hours | 1.30 | 1.08 - 1.58 | 0.006 |
| Interaction effects |  |  |  |
| *Pf*MSP1_19_: Age 6-15 years | 1.66 | 0.22 - 12.65 | 0.622 |
| *Pf*MSP1_19_: Age >15 years | 0.64 | 0.09 - 4.66 | 0.659 |

**Table S26. Odds of asymptomatic Pf infection amongst PfMSP1_19_ seropositive individuals.** Odds of asymptomatic infection if sero-positive to PfMSP1_19_ is adjusted for individual’s age group and LLIN use in the last 24 hours, allowing for interaction between sero-positivity and age group.

| Outcome: Asymptomatic *Pf* infection (detected by PCR) | | | |
| --- | --- | --- | --- |
|  | **Adjusted** | | |
|  | **aOR** | **95%CI** | **p-value** |
| *Pf*MSP1_19_ positive individual | 4.52 | 2.57 - 7.94 | <0.001 |
| Age group |  |  |  |
| 1-5 years | 1.00 | 1.00 | - |
| 6-15 years | 1.42 | 1.04 - 1.92 | 0.025 |
| > 15 years | 0.97 | 0.65 - 1.44 | 0.888 |
| LLIN use 24 hours | 1.00 | 0.96 - 1.05 | 0.950 |
| Interaction effects |  |  |  |
| *Pf*MSP1_19_: Age 6-15 years | 0.52 | 0.29 - 0.93 | 0.028 |
| *Pf*MSP1_19_: Age >15 years | 0.48 | 0.26 - 0.88 | 0.017 |

**Table S27. Odds of clinical malaria amongst PfAMA1 seropositive individuals and compounds.** Odds of clinical malaria if an individual is concurrently sero-positive to PfAMA1 is shown as unadjusted and adjusted for individual’s age group and LLIN use in the last 24 hours. Odds of clinical malaria if an individual is residing in the same compound as a PfAMA1 sero-positive individual is shown as unadjusted and adjusted for individual’s age group and compound size. Odds of clinical malaria based on whether compound sero-prevalence to PfAMA1 is <50% or >50% is shown unadjusted and adjusted for individual’s age group and LLIN use in the last 24 hours.

| Outcome: Clinical infection (passive case detection or fever and RDT+) | | |  |  |  |  |
| --- | --- | --- | --- | --- | --- | --- |
|  | **Unadjusted** | | | **Adjusted** | | |
|  | **OR** | **95%CI** | **p-value** | **OR** | **95%CI** | **p-value** |
| *Pf*AMA1 positive individual | 2.21 | 1.25 - 3.90 | 0.006 | 2.32 | 1.40 - 3.85 | 0.001 |
| Age group |  |  |  |  |  |  |
| 1-5 years | - | - | - | 1.00 | 1.00 | - |
| 6-15 years | - | - | - | 2.02 | 0.91 - 4.48 | 0.083 |
| > 15 years | - | - | - | 0.63 | 0.27 - 1.45 | 0.277 |
| LLIN use 24 hours | - | - | - | 1.39 | 1.04 - 1.85 | 0.024 |
| Residing in compound with sero-positive individual | 0.06 | 0.01 - 0.62 | 0.018 | 0.05 | 0.01 - 0.52 | 0.011 |
| Age group (individual) | - | - | - | 1.49 | 0.44 - 5.07 | 0.526 |
| *Pf*AMA1 sero-prevalence compound |  |  |  |  |  |  |
| Sero-prevalence <50% | 0.18 | 0.03 - 1.31 | 0.091 | 0.18 | 0.03 - 1.08 | 0.061 |
| Sero-prevalence >50% | 0.62 | 0.07 - 5.66 | 0.674 | 0.74 | 0.09 - 6.31 | 0.783 |
| Age group (individual) | - | - | - | 1.12 | 0.65 - 1.95 | 0.680 |
| LLIN use 24 hours | - | - | - | 2.28 | 0.59 - 8.77 | 0.230 |

**Table S28. Odds of asymptomatic Pf infection amongst PfAMA1 seropositive individuals and compounds.** Odds of asymptomatic Pf infection if an individual is concurrently sero-positive to PfAMA1 is shown as unadjusted and adjusted for individual’s age group and LLIN use in the last 24 hours. Odds of asymptomatic Pf infection if an individual is residing in the same compound as an PfAMA1 sero-positive individual is shown as unadjusted and adjusted for individual’s age group and compound size. Odds of asymptomatic infection based on whether compound sero-prevalence to PfAMA1 is <50% or >50% is shown unadjusted and adjusted for individual’s age group and LLIN use in the last 24 hours.

| Outcome: Asymptomatic *Pf* infection (detected by PCR) | | | | | |  |
| --- | --- | --- | --- | --- | --- | --- |
|  | **Unadjusted** | | | **Adjusted** | | |
|  | **OR** | **95%CI** | **p-value** | **aOR** | **95%CI** | **p-value** |
| *Pf*AMA1 positive individual | 2.62 | 2.12 - 3.24 | <0.001 | 3.80 | 2.95 - 4.90 | <0.001 |
| Age group |  |  |  |  |  |  |
| 1-5 years | - | - | - | 1.00 | 1.00 | - |
| 6-15 years | - | - | - | 0.97 | 0.73 - 1.30 | 0.862 |
| > 15 years | - | - | - | 0.47 | 0.33 - 0.69 | <0.001 |
| LLIN use 24 hours | - | - | - | 1.01 | 0.96 - 1.07 | 0.582 |
| *Pf*AMA1 positive compound | 1.96 | 1.01 - 3.81 | 0.046 | 1.99 | 1.00 - 3.93 | 0.048 |
| Age group (individual) | - | - | - | 1.10 | 0.77 - 1.56 | 0.598 |
| *Pf*AMA1 sero-prevalence compound |  |  |  |  |  |  |
| Sero-prevalence <50% | 2.33 | 0.95 - 5.69 | 0.063 | 2.21 | 0.89 - 5.49 | 0.089 |
| Sero-prevalence >50% | 6.08 | 2.21 - 16.76 | <0.001 | 6.24 | 2.23 - 17.51 | <0.001 |
| Age group (individual) | - | - | - | 1.09 | 0.75 - 1.57 | 0.658 |
| LLIN use 24 hours | - | - | - | 0.98 | 0.93 - 1.04 | 0.502 |

**Table S29. Odds of clinical malaria amongst PfAMA1 seropositive individuals.** Odds of clinical malaria if sero-positive to PfAMA1 is adjusted for individual’s age group and LLIN use in the last 24 hours, allowing for interaction between sero-positivity and age group.

| Outcome: Clinical malaria (passive case detection or fever and RDT+) | | | |
| --- | --- | --- | --- |
|  | **Adjusted** | | |
|  | **aOR** | **95%CI** | **p-value** |
| *Pf*AMA1 positive individual | 5.08 | 0.90 - 28.69 | 0.066 |
| Age group |  |  |  |
| 1-5 years | 1.00 | 1.00 | - |
| 6-15 years | 4.48 | 1.71 - 11.70 | 0.002 |
| > 15 years | 0.65 | 0.18 - 2.33 | 0.508 |
| LLIN use 24 hours | 1.37 | 1.14 - 1.63 | 0.001 |
| Interaction effects |  |  |  |
| *Pf*AMA1: Age 6-15 years | 0.48 | 0.06 - 3.61 | 0.475 |
| *Pf*AMA1: Age >15 years | 0.80 | 0.10 - 6.73 | 0.837 |

**Table S30. Odds of asymptomatic Pf infection amongst PfAMA1 seropositive individuals.** Odds of asymptomatic infection if sero-positive to PfAMA1 is adjusted for individual’s age group and LLIN use in the last 24 hours, allowing for interaction between sero-positivity and age group.

| Outcome: Asymptomatic *Pf* infection (detected by PCR) | | | |
| --- | --- | --- | --- |
|  | **Adjusted** | | |
|  | **aOR** | **95%CI** | **p-value** |
| *Pf*AMA1 positive individual | 4.74 | 2.78 - 8.07 | <0.001 |
| Age group |  |  |  |
| 1-5 years | 1.00 | 1.00 | - |
| 6-15 years | 0.92 | 0.66 - 1.27 | 0.616 |
| > 15 years | 0.81 | 0.51 - 1.31 | 0.395 |
| LLIN use 24 hours | 1.01 | 0.96 - 1.06 | 0.629 |
| Interaction effects |  |  |  |
| *Pf*AMA1: Age 6-15 years | 0.99 | 0.55 - 1.77 | 0.966 |
| *Pf*AMA1: Age >15 years | 0.45 | 0.23 - 0.85 | 0.015 |

**Table S31. Odds of clinical malaria amongst PfGLURP.R2 seropositive individuals and compounds.** Odds of clinical malaria if an individual is concurrently sero-positive to PfGLURP.R2 is shown as unadjusted and adjusted for individual’s age group and LLIN use in the last 24 hours. Odds of clinical malaria if an individual is residing in the same compound as a PfGLURP.R2 sero-positive individual is shown as unadjusted and adjusted for individual’s age group and compound size. Odds of clinical malaria based on whether compound sero-prevalence to PfGLURP.R2 is <50% or >50% is shown unadjusted and adjusted for individual’s age group and LLIN use in the last 24 hours.

| Outcome: Clinical infection (passive case detection or fever and RDT+) | | |  |  |  |  |
| --- | --- | --- | --- | --- | --- | --- |
|  | **Unadjusted** | | | **Adjusted** | | |
|  | **OR** | **95%CI** | **p-value** | **OR** | **95%CI** | **p-value** |
| *Pf*GLURP.R2 positive individual |  |  |  |  |  |  |
| Age group | 2.17 | 1.28 - 3.68 | 0.004 | 3.12 | 2.12 - 4.59 | <0.001 |
| 1-5 years | - | - | - | 1.00 | 1.00 | - |
| 6-15 years | - | - | - | 1.96 | 0.91 - 4.18 | 0.084 |
| > 15 years | - | - | - | 0.48 | 0.23 - 1.04 | 0.063 |
| LLIN use 24 hours | - | - | - | 1.39 | 1.05 - 1.83 | 0.022 |
| Residing in compound with sero-positive individual | 0.06 | 0.01 - 0.53 | 0.012 | 0.05 | 0.01 - 0.43 | 0.006 |
| Age group (individual) | - | - | - | 1.53 | 0.44 - 5.34 | 0.502 |
| *Pf*GLURP.R2 sero-prevalence compound |  |  |  |  |  |  |
| Sero-prevalence <50% | 0.23 | 0.03 - 2.03 | 0.186 | 0.24 | 0.04 - 1.62 | 0.143 |
| Sero-prevalence >50% | 0.21 | 0.01 - 3.16 | 0.261 | 0.39 | 0.03 - 3.09 | 0.310 |
| Age group (individual) | - | - | - | 1.15 | 0.68 - 1.93 | 0.597 |
| LLIN use 24 hours | - | - | - | 2.21 | 0.64 - 7.69 | 0.213 |

**Table S32. Odds of asymptomatic Pf infection amongst PfGLURP.R2 seropositive individuals and compounds.** Odds of asymptomatic Pf infection if an individual is concurrently sero-positive to PfGLURP.R2 is shown as unadjusted and adjusted for individual’s age group and LLIN use in the last 24 hours. Odds of asymptomatic Pf infection if an individual is residing in the same compound as an PfGLURP.R2 sero-positive individual is shown as unadjusted and adjusted for individual’s age group and compound size. Odds of asymptomatic infection based on whether compound sero-prevalence to PfGLURP.R2 is <50% or >50% is shown unadjusted and adjusted for individual’s age group and LLIN use in the last 24 hours.

| Outcome: Asymptomatic *Pf* infection (detected by PCR) | | | | | |  |
| --- | --- | --- | --- | --- | --- | --- |
|  | **Unadjusted** | | | **Adjusted** | | |
|  | **OR** | **95%CI** | **p-value** | **aOR** | **95%CI** | **p-value** |
| *Pf*GLURP.R2 positive individual | 2.38 | 1.96 - 2.89 | <0.001 | 3.80 | 2.92 - 4.95 | <0.001 |
| Age group |  |  |  |  |  |  |
| 1-5 years | - | - | - | 1.00 | 1.00 | - |
| 6-15 years | - | - | - | 1.03 | 0.77 - 1.39 | 0.830 |
| > 15 years | - | - | - | 0.43 | 0.29 - 0.64 | <0.001 |
| LLIN use 24 hours | - | - | - | 1.01 | 0.96 - 1.06 | 0.636 |
| *Pf*GLURP.R2 positive compound | 1.59 | 0.85 - 2.97 | 0.145 | 1.52 | 0.77 - 3.01 | 0.228 |
| Age group (individual) | - | - | - | 1.10 | 0.77 - 1.56 | 0.598 |
| *Pf*GLURP.R2 sero-prevalence compound |  |  |  |  |  |  |
| Sero-prevalence <50% | 1.57 | 0.69 - 3.54 | 0.280 | 1.42 | 0.58 - 3.51 | 0.446 |
| Sero-prevalence >50% | 2.56 | 1.06 - 6.22 | 0.037 | 2.43 | 0.93 - 6.36 | 0.072 |
| Age group (individual) | - | - | - | 1.09 | 0.76 - 1.58 | 0.635 |
| LLIN use 24 hours | - | - | - | 0.99 | 0.93 - 1.04 | 0.592 |

**Table S33. Odds of clinical malaria amongst PfGLURP.R2 seropositive individuals.** Odds of clinical malaria if sero-positive to PfGLURP.R2 is adjusted for individual’s age group and LLIN use in the last 24 hours, allowing for interaction between sero-positivity and age group.

| Outcome: Clinical malaria (passive case detection or fever and RDT+) | | | |
| --- | --- | --- | --- |
|  | **Adjusted** | | |
|  | **aOR** | **95%CI** | **p-value** |
| *Pf*GLURP.R2 positive individual | 4.59 | 1.05 - 20.13 | 0.043 |
| Age group |  |  |  |
| 1-5 years | 1.00 | 1.00 | - |
| 6-15 years | 3.24 | 1.22 - 8.65 | 0.019 |
| > 15 years | 0.63 | 0.12 - 3.26 | 0.583 |
| LLIN use 24 hours | 1.34 | 1.12 - 1.61 | 0.002 |
| Interaction effects |  |  |  |
| *Pf*GLURP.R2: Age 6-15 years | 0.92 | 0.17 - 5.05 | 0.924 |
| *Pf*GLURP.R2: Age >15 years | 0.72 | 0.07 - 6.86 | 0.772 |

**Table S34. Odds of asymptomatic Pf infection amongst PfGLURP.R2 seropositive individuals.** Odds of asymptomatic infection if sero-positive to PfGLURP.R2 is adjusted for individual’s age group and LLIN use in the last 24 hours, allowing for interaction between sero-positivity and age group.

| Outcome: Asymptomatic *Pf* infection (detected by PCR) | | | |
| --- | --- | --- | --- |
|  | **Adjusted** | | |
|  | **aOR** | **95%CI** | **p-value** |
| *Pf*GLURP.R2 positive individual | 6.18 | 3.79 - 10.07 | <0.001 |
| Age group |  |  |  |
| 1-5 years | 1.00 | 1.00 | - |
| 6-15 years | 1.20 | 0.84 - 1.71 | 0.320 |
| > 15 years | 0.89 | 0.49 - 1.59 | 0.689 |
| LLIN use 24 hours | 1.01 | 0.96 - 1.06 | 0.676 |
| Interaction effects |  |  |  |
| *Pf*GLURP.R2: Age 6-15 years | 0.62 | 0.38 - 1.00 | 0.049 |
| *Pf*GLURP.R2: Age >15 years | 0.32 | 0.16 - 0.64 | 0.001 |
