# Supplementary material for: Antibody responses to a suite of novel serological markers for malaria surveillance demonstrate strong correlation with clinical and parasitological infection across seasons and transmission settings in The Gambia
Source: BMC Med. 2020 Sep 25;18:304. doi: 10.1186/s12916-020-01724-5 (PMC7517687; doi:10.1186/s12916-020-01724-5)
Supplement: Supplementary file 3 — Additional file 3. Map of household geolocation of PfAMA1 sero-positive, clinical malaria and Pf infections across four villages during dry and wet transmission seasons. Spatial distribution of infections shown for lower transmission (N’demban) and higher transmission (Besse) villages in the West Coast Region (WCR) and lower transmission (Njaiyal) and higher transmission (Madina Samako) villages in the Upper River Region (URR). Infections at the start of the wet season (June – July 2013) shown on the left and during the wet and transmission season (August – December 2013) on the right. [file 12916_2020_1724_MOESM3_ESM.docx]

**Figure S2. Map of household geolocation of *Pf*AMA1 sero-positive, clinical malaria and *Pf* infections across four villages during dry and wet transmission seasons.** Spatial distribution of infections shown for lower transmission (N’demban) and higher transmission (Besse) villages in the West Coast Region (WCR) and lower transmission (Njaiyal) and higher transmission (Madina Samako) villages in the Upper River Region (URR). Infections at the start of the wet season (June – July 2013) shown on the left and during the wet and transmission season (August – December 2013) on the right.

**
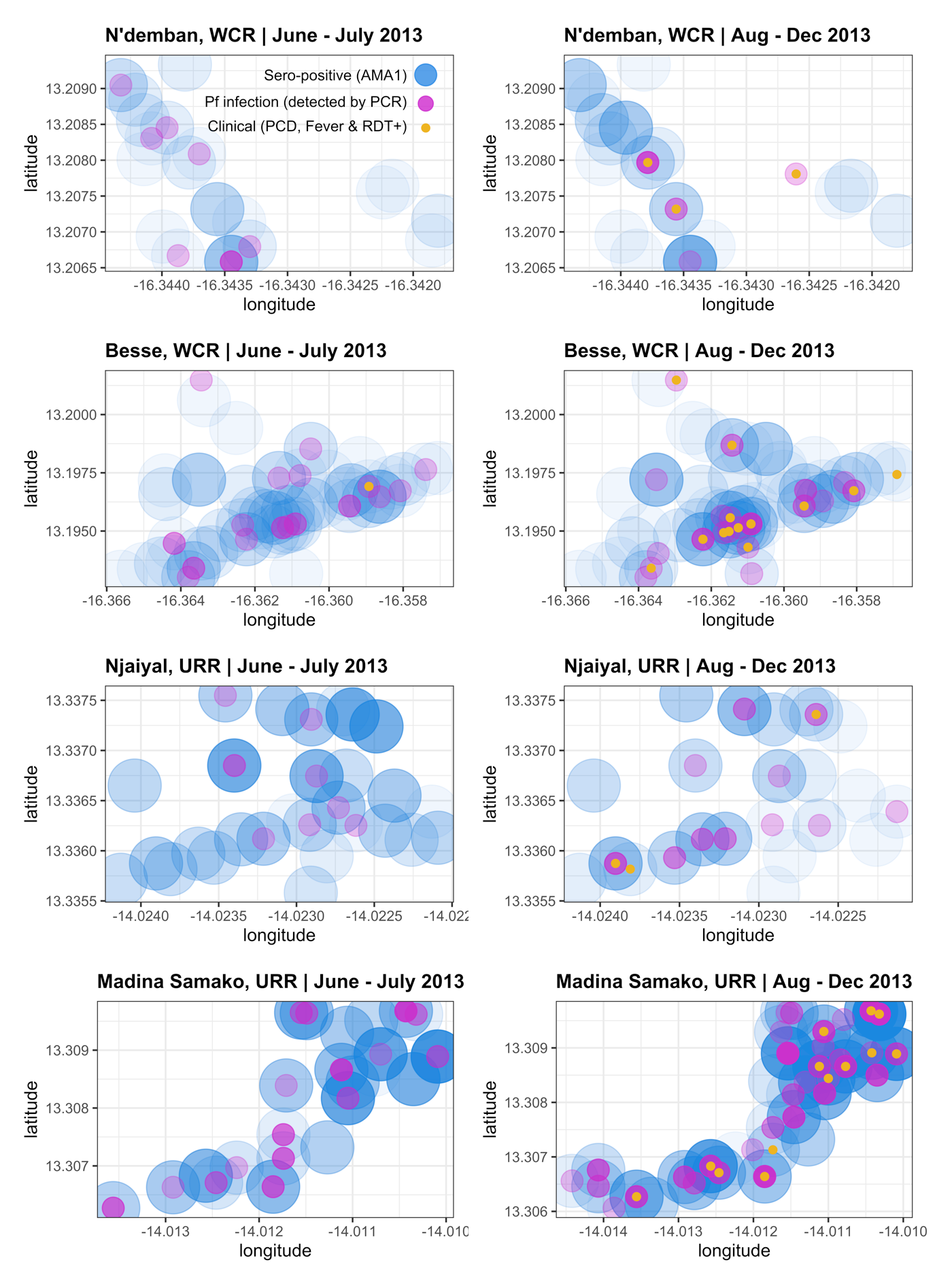
**

**Figure S2. Map of household geolocation of *Pf*AMA1 sero-positive, clinical malaria and *Pf* infections across four villages during dry and wet transmission seasons.** Spatial distribution of infections shown for lower transmission (N’demban) and higher transmission (Besse) villages in the West Coast Region (WCR) and lower transmission (Njaiyal) and higher transmission (Madina Samako) villages in the Upper River Region (URR). Infections at the start of the wet season (June – July 2013) shown on the left and during the wet and transmission season (August – December 2013) on the right.
